# Rhizosphere microbes enhance plant salt tolerance: Toward crop production in saline soil

**DOI:** 10.1016/j.csbj.2022.11.046

**Published:** 2022-11-25

**Authors:** Yunpeng Liu, Weibing Xun, Lin Chen, Zhihui Xu, Nan Zhang, Haichao Feng, Qiang Zhang, Ruifu Zhang

**Affiliations:** a Key Laboratory of Microbial Resources Collection and Preservation, Ministry of Agriculture and Rural Affairs, Institute of Agricultural Resources and Regional Planning, Chinese Academy of Agricultural Sciences, Beijing 100081, PR China; b Jiangsu Provincial Key Lab for Organic Solid Waste Utilization, National Engineering Research Center for Organic-based Fertilizers, Jiangsu Collaborative Innovation Center for Solid Organic Waste Resource Utilization, Nanjing Agricultural University, Nanjing 210095, PR China; c Experimental Center of Forestry in North China, Chinese Academy of Forestry, Beijing 102300, PR China; d Heze Kingenta Ecological Engineering Co., Ltd, Heze, Shandong 274000, PR China

**Keywords:** Plant salt tolerance, Rhizosphere microbe, Saline soil, Microbe-enhanced salt tolerance mechanisms

## Abstract

The world’s population continues to increase and thus requires more food production to take place in nonarable land, such as saline soil; therefore, it is urgent to find solutions to enhance the salinity tolerance of crops. As the second genome of plants, the rhizosphere microbiome plays critical roles in plant fitness under stress conditions. Many beneficial microbes that help plants cope with salinity stress have been identified, highlighting their roles in mitigating salt stress-induced negative effects on plants. However, a comprehensive review of the microbial species that are able to confer plant salt tolerance and the underlying mechanisms is still lacking. In this review, we compared the representative fungal and bacterial taxa that demonstrate the ability to enhance plant growth in saline soil. We also reviewed the mechanisms by which rhizosphere microbes enhance plant salt stress tolerance, i.e., by re-establishing ion and osmotic homeostasis, preventing damage to plant cells, and resuming plant growth under salt stress. Finally, future research efforts to explore the rhizosphere microbiome for agricultural sustainability are proposed.

## 1 Introduction

Agricultural production worldwide is facing the challenge of increasing population size. Food production is increasingly restricted by limited cultivated land due to soil degradation issues, such as salinization. Saline soil accounts for 6 % of the world’s total land area and is a severe hindrance to agricultural production [1]. Most domesticated crops are salt sensitive and cannot be cultivated in saline soil [2]. Currently, the increasing food demand of global populations makes finding a solution to enhance crop tolerance to salinity more urgent.

As the functions of rhizosphere microbes in plant fitness have been recognized [3], many beneficial microbes that help plants cope with salinity stress have been isolated and reapplied to the soil to enhance crop production in saline soil. These microbes can be used in different ways; the common method is to make microbial fertilizers or microbial agents [4], [5], which can be applied to the soil directly or combined with chemical or organic fertilizers. In addition, some strains can be coated on crop seeds before sowing [6]. Their efficiency in increasing plant growth or promoting seed germination under saline conditions has been shown, indicating a role in the mitigation of salt stress-induced negative effects on plants. The plant salt tolerance induced by some microbial groups, such as bacterial endophytes [7], the halophyte microbiome [8], microbial 1-aminocyclopropane-1-carboxylic acid (ACC) deaminase [9] or microbial coinoculation [10], has been reviewed, as well as the microbial metabolites responsible for enhancing plant salt tolerance [11]. However, a comprehensive comparison of the microbial species that are able to confer plant salt tolerance and the underlying mechanisms is still lacking.

Here, we reviewed the fungal and bacterial isolates with clear evidence of promoting plant growth or seed germination under saline stress. Specifically, we discussed the challenges for the agricultural application of each kind of microbial inoculant. We also summarized the mechanisms by which microbes enhance plant tolerance to salt stress. The salt stress signaling and tolerance mechanism of plants have been well reviewed [2], [12], [13], [14], [15], [16], [17], [18] and will not be included in this review.

## 2 Microbial taxa enhancing plant tolerance to salt stress

### 2.1 Arbuscular mycorrhizal fungi (AMF)

AMF are well-known beneficial fungi belonging to the phylum Glomeromycota that have close symbiotic interactions with their host plants. The main function of AM symbiosis seems to be assisting plants with nutrient uptake by extending the active absorption area of roots [10], [19]. Several studies have also demonstrated the effect of AMF on plant productivity under salt stress. AMF, including the genera *Glomus*, *Funneliformis*, *Septoglomus*, *Gigaspora*, *Acaulospora*, *Claroideoglomus*, and *Rhizophagus* (*Funneliformis*, *Claroideoglomus* and *Rhizophagus* used to belong to *Glomus*), have shown the capability to enhance plant salt tolerance (Table S1). The plants that showed enhanced production under saline stress upon AMF inoculation include lettuce (up to 80 mM NaCl) [20], maize (up to 100 mM NaCl) [21], wheat (up to 200 mM NaCl) [22], rice (up to 120 mM NaCl) [23] and some leguminous (up to 200 mM NaCl) [24] and woody plants (Table S1). The characteristics and advances of AMF in mitigating plant salt stress include integrity with significantly enhanced plant nutrient uptake, such as nitrogen (N) and potassium (K), especially phosphorus (P) [25]. AMF-induced plant salt tolerance is always correlated with phosphorus, which plays an important role in photosynthesis to supply energy for the stress response [26]. However, the symbiosis of AMF with plants is negatively affected by salt stress because AMF are not salt tolerant, which has been confirmed by several studies [25], [27], [28], [29], [30]. Pandey et al. [31] found that native AMF isolated from saline soil are more effective in mitigating the negative effect of salt stress on *Cajanus cajan*, suggesting that the salt tolerance of AMF themselves is also a limiting factor for performance under salt stress.

### 2.2 *Trichoderma* spp.

*Trichoderma* spp. are free-living soil filamentous fungi that are beneficial to plants [32]. *Trichoderma* strains are nonsymbiotic, while some strains can infect root outer cells [32]. *Trichoderma* has diverse beneficial functions for plants, such as enhancing plant stress resistance and promoting plant growth. *T. harzianum, T. virens, T. atroviride, T. asperelloides, T. longibrachiatum, T. yunnanense,* and *T. afroharzianum* have shown the ability to promote seed germination and plant growth of *Arabidopsis* (up to 100 mM NaCl) [33], *cucumber* (up to 200 mM NaCl) [34], *wheat* (up to 200 mM NaCl) [6] and *barley* (up to 200 mM NaCl) [35] under salt stress (Table S1). These strains promoted root development and affected root system architecture. In addition, most *Trichoderma* strains isolated from soil are efficient decomposers of dead plant residues that drive soil carbon cycling and plant growth [32]. *Trichoderma* strains are not an outstanding microbial group regarding salt tolerance when compared with some bacterial strains, such as *Bacillus* spp. and *Enterobacter* spp. Contreras-Cornejo et al. [36] demonstrated that 200 mM NaCl significantly inhibited the hyphal growth of *T. virens* and *T. atroviride*. Zhang et al. [37] found similar results that 30 mg/mL NaCl inhibited the growth of *Trichoderma longibrachiatum* T6. Therefore, both salt tolerance for itself and plant salt tolerance enhancement are necessary for the successful cultivation of a good functional *Trichoderma* isolate in saline soil.

### 2.3 Serendipita indica (formerly *Piriformospora indica*)

*Piriformospora indica* is a natural soil-inhabiting filamentous fungus that promotes plant growth upon symbiosis. *P. indica* is an endophyte for a broad range of host plants (Franken 2012). It was first isolated from an AMF spore by Verma et al. in 1998. However, unlike AMF, *P. indica* can be cultivated on defined media. Several reviews have concluded the beneficial effects of *P. indica* on plants [10], [38], [39], [40]. In recent years, *P. indica* has shown impressive potential in mitigating salt stress in *Arabidopsis* (up to 200 mM NaCl) [41], *rice* (up to 300 mM NaCl) [42], *barley* (up to 300 mM NaCl) [43] and *tomato* (up to 150 mM NaCl) [44] (Table S1). Lanza et al. [45] demonstrated that free-living *P. indica* is sensitive to salt; it can only tolerate up to 100 mM NaCl, and hyphal growth was significantly inhibited due to the toxic effect of Na^+^ rather than osmotic stress. Interestingly, Vahabi et al. [41] demonstrated that the growth promotion of Arabidopsis seedlings under salt stress is strongly dependent on physical contact between the two symbionts.

### 2.4 *Enterobacter* spp.

The specific role of *Enterobacter* spp. as beneficial endophytes in enhancing plant salt tolerance has been demonstrated in their interactions with *Arabidopsis* (up to 200 mM NaCl) [46], *rice* (up to 150 mM NaCl) [47], *wheat* (up to 200 mM NaCl) [48], *maize* (electrical conductivity (EC) up to 13.6 dS/m) [49], *tomato* (up to 200 mM NaCl) [46], *mung bean* (1 % NaCl) [50], *okra* (75 mM NaCl) [51] and *quinoa* (400 mM NaCl) [52] (Table S1). *Enterobacter* spp. were first recognized as beneficial plant bacteria due to ACC deaminase activity [53]. *Enterobacter* sp. SA187 is a well-studied isolate that promotes plant growth under salt stress. It seems that the modulation of ethylene signaling in plants plays a key role in SA187-induced salt tolerance [54]. Ali et al. [55] demonstrated that *E. cloacae* PM2 can survive under salinity stress of up to 3 M NaCl in Luria-Bertani (LB) medium. However, the careful selection of plant-safe strains is necessary given that members of the *Enterobacter* genus are generally known as opportunistic pathogens of plants and humans [56].

### 2.5 *Bacillus* spp.

*Bacillus* spp. are gram-positive spore-forming bacteria from the phylum *Firmicutes* and represent the most widely used and well-studied bacteria in agricultural production. Most of the *Bacillus* strains that are beneficial to plants belong to the *subtilis* subgroup. These bacteria are generally root surface colonizers or root endophytes that exert diverse plant beneficial functions. *Bacillus* spp. have been reported to induce salt tolerance in *Arabidopsis* (up to 250 mM NaCl) [57], [58], *maize* (up to 100 mM NaCl) [59], *barley* (up to 200 mM NaCl) [60], *rice* (up to 200 mM NaCl) [61], wheat (up to 400 mM NaCl) [62], tomato (up to 300 mM NaCl) [63], chickpea (2 % NaCl) [64], cotton (up to 200 mM NaCl) [65], soybean (up to 240 mM NaCl) [66] and some halophytes (Table S1). *Bacillus* can tolerate high salt concentrations and form stress-tolerant spores when exposed to adverse environments [55]. The feature of spore formation greatly increases bacterial survival in soil.

### 2.6 *Pseudomonas* spp.

*Pseudomonas* spp. are gram-negative bacteria from the phylum *Proteobacteria* and represent one of the most abundant rhizobacterial groups. The species enhancing plant salt tolerance include *P. putida, P. simiae, P. syringae, P. fluorescens, P. migulae, P. thivervalensis, P. geniculate, P. corrugate, P. stutzeri, P. chlororaphis, P. extremorientalis, P. extremorientalis, P. aurantiaca* and *P. chlororaphis*. *Arabidopsis* (up to 200 mM NaCl) [67], *maize* (up to 150 mM NaCl) [68], *wheat* (up to 200 mM NaCl) [69], *cotton* (0.35 % NaCl) [70], *cucumber* (100 mM NaCl) [71], *peanut* (100 mM NaCl) [72], *soybean* (100 mM NaCl) [73], [74], *tomato* (up to 185 mM NaCl) [75], *sunflower* (EC up to 15.9 dS/m) [76], *oats* and *barley* (EC up to 9.4 dS/m) [77] have shown elevated growth under salt stress upon inoculation with *Pseudomonas* spp. (Table S1). *Pseudomonas* is a naturally highly abundant bacterial group in the rhizosphere, suggesting that *Pseudomonas* is adapted to the rhizosphere and may perform well in colonizing roots and thus enhancing plant salt tolerance. However, as nonspore-forming bacteria, the relatively low survival rate of microbial fertilizers or microbial agents during the process of production and storage may be disadvantageous for their application in agriculture.

## 3 Mechanisms by which microbes enhance plant salt tolerance

High salinity is a complex type of stress for plants. On the one hand, high amounts of Na^+^ and Cl^−^ in the cytoplasm (mainly Na^+^) are directly toxic to cells, as they interfere with many protein functions, thus influencing cell physiology [2], [16]. On the other hand, high salt concentrations in soil lead to hyperosmotic stress in plant cells and subsequent difficulty in acquiring water and soil nutrients [2], [16]. Both direct ion stress and osmotic stress can be sensed immediately after plants are exposed to high-salinity conditions. After perceiving the primary signal, secondary signals, such as Ca^2+^, ROS and ABA, are generated to induce stress-responsive genes [18]. However, if ROS accumulate, the cells are damaged, and programmed cell death is initiated. ABA accumulation induces stomatal closure to reduce transpiration, which also reduces water use efficiency (WUE) and CO_2_ use efficiency (CUE). To counter the negative effects of Na^+^ and ROS causing cell damage and reducing WUE and CUE, plants must produce more ROS-scavenging enzymes, transport Na^+^ into vacuoles, and produce osmolytes, which are costly with regard to energy, thereby leading to growth inhibition [1], [13], [17], [18].

**Fig. 1:**
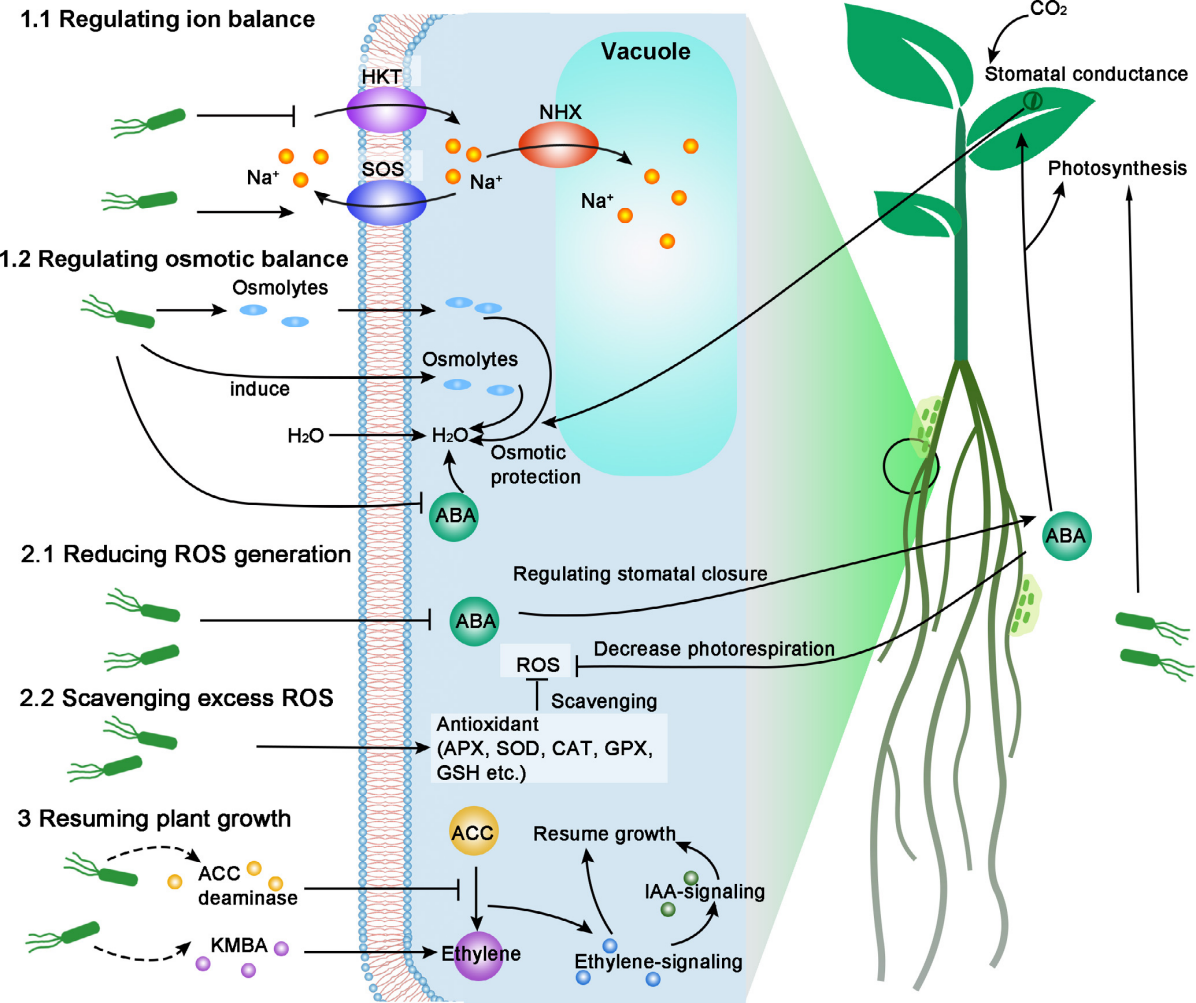
Mechanisms by which microbes enhance plant salt tolerance. 1. To re-establish the ion balance of plants under salt stress, microbes reduce the Na^+^ content in the cytoplasm by decreasing Na^+^ uptake, increasing Na^+^ efflux and comparting Na^+^ into vacuoles. To regulate the osmotic balance of plants under salt stress, microbes produce osmolytes and induce plants to produce osmolytes, and they also reduce ABA to increase water uptake. 2. To prevent ROS-induced damage to plants under salt stress, microbes can reduce plant ROS generation and stimulate plants to scavenge ROS. 3. To resume plant growth under salt stress, microbes promote photosynthesis and produce ACC deaminase or other compounds to regulate plant ethylene signaling.

The NaCl tolerance of microbes far exceeds the salt tolerance of plants. Microbes can concentrate compatible solutes to protect against the osmotic imbalance between the cytoplasm and the environment and express diverse Na^+^ transporters to reduce cytoplasmic Na^+^ concentrations. In addition to tolerating high salinity, many fungal and bacterial strains can enhance plant tolerance to salinity and mitigate the salt stress-induced inhibition of plant growth. Resuming growth under stress conditions is risky because growth inhibition by stress physiology is actually an energy trade-off with tolerance [16]. Nevertheless, microbes have multiple effects on plant ion and osmotic balance, ROS regulation and detoxification, and photosynthesis. These processes are interrelated and ultimately promote plant growth under salt stress via three mechanisms: re-establishing ion and osmotic homeostasis, preventing damage to plant cells, and resuming plant growth under salt stress (Fig. 1).

### 3.1 Re-establishing ion and osmotic homeostasis

#### 3.1.1 Ion balance

The accumulation of Na^+^ in the plant cytoplasm is toxic to plant cells. Na^+^ competes with other ions to bind proteins, which leads to the inactivation of enzymes. Na^+^ also inhibits the uptake of K^+^ and other ions due to the impact of ion balance between the two sides of the cytoplasmic membrane. Therefore, the accumulation of Na^+^ in the cytoplasm must be reduced, and the ion balance must be re-established for roots to acquire other ions.

To counter the high Na^+^ concentration in the environment, plants need to reduce Na^+^ influx through the plasma membrane, which is mediated by the high-affinity K^+^ channel (HKT) [78], [78], [79]. Many microbial strains have been shown to increase plant shoot and/or root K^+^/Na^+^ ratios by increasing K^+^ and/or reducing Na^+^ in the cytoplasm, but the underlying mechanisms vary. HKT1, which plays critical roles in both Na^+^ influx across the plasma membrane and long-distance Na^+^ transport, is considered a key determinant of plant salt tolerance [79]. Expression of HKT1 in shoots promotes plant salt tolerance by reducing Na^+^ accumulation in shoots [80]. The specific role of HKT1 in the decrease in microbe-mediated plant Na^+^ under salt stress has been recognized. For example, Zhang et al. [81] demonstrated that *B. subtilis* GB03 stimulated HKT1 expression in shoots while reducing it in roots under 100 mM NaCl by producing VOCs. *Athkt1*;1, but not *sos3,* mutant plants failed to show GB03-induced plant growth and a decrease in Na^+^ under salt stress [81]. Niu et al. [82] demonstrated that enhanced *Puccinellia tenuiflora* salt tolerance is dependent on modulating the expression of *HKT1*. In contrast, *Pseudomonas simiae* AU decreased the expression of *HKT1* in soybean shoots under salt stress [73]. *P. indica* colonized *Arabidopsis* roots under salt stress and showed enhanced expression of HKT1[83]. Inoculation with AMF significantly induced HKT1;1 and HKT1;6 in both the roots and shoots of lettuce under 60 mM NaCl, but in the same study, inoculation with another AMF strain, *Claroideoglomus lamellosum*, did not significantly change the expression profiles for HKT1;1 and HKT1;6 in *lettuce* [84].

The excess Na^+^ in the cytoplasm under salt stress can be either exported out of the protoplast by the plasma membrane Na^+^/H^+^ antiporter [17] or compartmentalized into the vacuole by the vacuolar Na^+^/H^+^ exchanger (NHX) [85]. Increasing the Na^+^ efflux by the SOS pathway and vacuolar NHX-driven vacuolar Na^+^ compartmentalization is another strategy for reducing cytoplasmic Na^+^ accumulation [86]. SOS1, a plasma membrane Na^+^/H^+^ antiporter controlled by the regulation cascade of SOS2 and SOS3, is believed to be a key determinant of Na^+^ exclusion in roots [17]. Chen et al. [87], [88] showed that *B. amyloliquefaciens* SQR9 strongly stimulated the expression of *NHX1*, *NHX2* and *NHX3* in maize under salt stress by producing spermidine. However, either mutation of *NHX1* or blocking the SOS pathway, but not mutation with *HKT1*, leads to deficiency in SQR9- and spermidine-induced salt tolerance, indicating the determinant role of NHX1 and SOS1 in SQR9-induced salt tolerance [88]. Baek et al. [89] demonstrated a similar conclusion that the SOS pathway is necessary for *B. oryzicola* YC7007-induced Arabidopsis tolerance to salt stress. There are also some cases in which microbial inoculation increases K^+^ uptake but has no effect on Na^+^ accumulation [54].

#### 3.1.2 Osmotic balance

Soil salinity is always accompanied by hyperosmotic stress in plants. A high salt concentration is hyperosmotic to plant cells and will affect root water absorption or even water loss. This situation also limits the uptake of nutrients by roots and results in ABA accumulation. ABA signaling will lead to stomatal closure, which restricts transpiration and thus makes it harder to absorb water and reduce photosynthesis. Accumulating soluble osmoprotectants in the cytoplasm is a general strategy to counter hyperosmotic stress. These osmolytes, including charged metabolites, polyols, soluble sugars and complex sugars, vary based on plant species [2].

Most of the microbes that show mitigation effects on plant salt stress also increase the WUE and stimulate the production of different kinds of osmolytes by plants. The production of osmolytes under saline conditions can be controlled by microbes. For example, *Bacillus subtilis* GB03 produced VOCs that induced glycine betaine production by Arabidopsis under saline conditions in an ABA-dependent manner [81]. The mutation of trehalose synthesis in *Pseudomonas putida* UW4 completely blocked the promotion of tomato root development and growth under saline conditions, suggesting a critical role of microbe-produced osmolytes in plant salt tolerance [90], [91]. Salt stress-induced ABA accumulation is believed to be a signature of the stress response and contributes to stomatal closure [92]. Although ABA and proline accumulation in salt-stressed plants generally occurs, inoculation with microbes has inconsistent effects on ABA and proline contents under salt stress. While most studies propose that the observed ABA reduction in plants upon microbial inoculation is responsible for restoration of the osmotic balance under salt stress, *Bacillus licheniformis* SA03 [93], *Paenibacillus yonginensis* DCY84T [94] and *Bacillus mesonae* H20-5 [95] increased the ABA contents in *Chrysanthemum*, *Panax ginseng* and *tomato* under saline conditions, respectively. By inhibiting ABA synthesis using fluridone, Zhou et al. [93] demonstrated the key role of SA03-induced ABA in enhancing plant salt tolerance. The effects of microbial inoculations on plant proline accumulation upon saline stress also vary, and the physiological significance of proline accumulation in salt-stressed plants is controversial (Table S1). Proline protects plants from saline stress by increasing the osmosis of the cytoplasm and is induced upon salt stress and strengthened by microbial inoculation. However, a decrease in proline in inoculated plants compared with that of noninoculated plants under saline conditions has also been widely reported [53], [96], [97], [98]. Proline is an osmolyte and a signature of the stress response. Increased levels of proline upon microbial inoculation undoubtedly benefit salt tolerance, but the decreased levels of proline upon inoculation can also reflect a decrease in salt stress [99]. Therefore, the observed inconsistency in proline accumulation may also be due to the difference in the stress period. As plant endogenous osmolytes, proline and poly-γ-glutamic acid produced by microbes can also protect plants directly or act as a signal to induce plant salt tolerance [100], [101].

#### 3.2 Preventing damage to plant cells

Stress signaling following Ca^2+^ influx is always accompanied by ROS production [102]. ROS act as critical signals by activating various secondary messengers in the stress response, but they are also deleterious to plant cells by impairing DNA, proteins and lipids when they accumulate in plant cells [103]. The cell damage caused by ROS, including superoxide anion, hydrogen peroxide, hydroxyl radical, and singlet oxygen, is irreversible, especially for the elicitation of programmed cell death when hydroxyl radicals are produced [103]. Under normal conditions, ROS production and elimination are balanced; however, salt stress causes an imbalance due to the increase in ROS [102]. It is widely reported that plant electrolyte leakage and lipid peroxidation, as signatures of plasma membrane impairment by ROS [102], could be reduced by microbes.

##### 3.2.1 Reducing ROS generation

Inoculation with microbes that reduce ROS generation may exert beneficial effects in preventing or arresting oxidative damage. ROS are produced from two sources: metabolic ROS are mainly produced in the chloroplast, mitochondria and peroxisome via different pathways, and signaling ROS are produced in the apoplast by NADPH oxidases [104]. Under salt stress, there is a decrease in transpiration rates, which reduces CO_2_ availability to the plants. Salt stress also exposes chloroplasts to high excitation energy, which causes ROS generation [105]. It is evident that microbial inoculation is capable of stimulating stomatal conductance and increasing the CO_2_ availability of plants under saline conditions. In particular, increasing chlorophyll and carotenoid contents in salt-stressed plants is a common feature of microbe-primed plant salt tolerance (Table S1). Native AMF-inoculated maize showed higher efficiencies of photosystem II and stomatal conductance, which are believed to decrease photorespiration and ROS production [106], [107]. Sulfur metabolism is connected to photosynthesis due to its role in forming iron-sulfur cluster proteins, which act as electron carriers in photosynthesis. Andres-Barrao et al. [108] found that *Enterobacter* sp. SA187 suppressed Arabidopsis salt stress-induced ROS accumulation in plastids by increasing root sulfur assimilation. Inoculation with SA187 also rescued the hypersensitivity of Arabidopsis LSU mutants, in which four genes encoding chloroplast-targeted proteins (LSU1-LSU4) that activate Fe-superoxide dismutase were silenced [108].

##### 3.2.2 Scavenging excess ROS

In addition to reducing plant ROS generation, microbes can stimulate the ROS detoxification system in plants. Both the enzymatic antioxidant system, including superoxide dismutase (SOD), ascorbate peroxidase (APX), catalase (CAT), glutathione peroxidase (GPX), and peroxiredoxin (PRX), and the nonenzymatic system, including ascorbic acid, glutathione (GSH) and proline, are induced when plants are under salt stress; microbial inoculation can strengthen this induction process and stimulate plants to scavenge excess ROS. For example, Waller et al. [43] found that *P. indica*-infested roots under saline conditions increased the dehydroascorbate reductase activity and ascorbate content and decreased the level of dehydroascorbate. The critical role of stimulating plant ROS scavenging is confirmed by the fact that the salt tolerance induced by *B. velezensis* and the produced spermidine is dependent on plant GSH production [88]. It is evident that *Pseudomonas*-produced phenazine also contributes to catalase induction and thus ROS scavenging in plants under saline stress [69]. Moreover, some genes involved in Fe acquisition were activated in *Bacillus licheniformis* SA03-inoculated plants under salt stress [93]. Considering the Fenton reaction between iron and H_2_O_2_ that generates hydroxyl radicals [104], the modulation of iron homeostasis in plant cells by microbial inoculants may also contribute to the avoidance of hydroxyl radicals causing cell damage and subsequent cell death.

In contrast, several microbes with high antioxidant activity attenuated the salt stress-induced antioxidant system. For example, inoculation with *Brevibacterium linens* RS16 was found to reduce the salt stress-induced plant emissions of all major classes of volatile organic compounds (VOCs), which serve as antioxidants to tolerate stress [109]. An endophytic isolate of the fungus *Yarrowia lipolytica* selected on the basis of high antioxidant activity has reduced the peroxidase and catalase activity levels of maize in saline conditions compared to noninoculated maize [97]. These examples suggest that microbes can also enhance the salt tolerance of plants by directly scavenging ROS, which results in a reduction in stress-induced antioxidant production in plants.

#### 3.3 Resuming plant growth under salt stress

Transporting ions, producing osmolytes and scavenging ROS, as discussed above, are all energy-consuming processes. For plants under salt-stressed conditions, resuming growth is a difficult choice for energy distribution and is precisely controlled by a very complex signaling transduction network.

##### 3.3.1 Promotion of photosynthesis

An important determinant of microbe-induced salt tolerance is the improvement of photosynthesis to compensate for the energy cost of tolerance. It is generally recognized that salt stress greatly reduces the chlorophyll content, stomatal conductance and intercellular CO_2_ concentration and strongly inhibits the net photosynthetic and transpiration rates [110]. As discussed above, almost all microbe-induced plant growth under salinity stress occurs in conjunction with the mitigation of stress-reduced photosynthesis. Increased biomass is always correlated with the net photosynthetic rate (Table S1). Increased transpiration by microbe inoculation will then lead to higher water absorption and nutrient uptake by roots, including those of phosphorus and sulfur, and thus promote photosynthesis. There is a possibility that the increased water absorption and nutrient uptake in stressed plants upon microbe inoculation is also a result of the microbial manipulation of the root system architecture, which occurs commonly in plant–microbe interactions [36]. The mechanism by which microbes initiate this positive feedback in plants under saline conditions is not clear. Since microbes can always directly or indirectly influence plant hormone signaling, which controls photosynthesis, it is necessary to understand the roles of phytohormones in microbe-induced plant growth under saline conditions.

##### 3.3.2 Modulating IAA and ethylene signaling

IAA is important for plant growth and root development but is not a generally known central phytohormone in salt-stress responses in plants [111]. Salt stress-induced plant growth inhibition results from the suppression of IAA signaling, as shown by the decreased GUS density in the *DR5:uidA* line and the severely reduced fresh weight of auxin transport or signaling mutants, such as *arf7arf19* and *tir1afb2afb3,* under saline conditions [36]. Several studies have demonstrated the microbial functions in activating IAA signaling and increasing endogenous IAA content in salt-stressed plants [36], [37], [70], [112]. IAA signaling has been proven to play a key role in *Trichoderma*- and *Bacillus*-modulated root systemic architecture (RSA) under nonstress conditions since mutations in genes involved in auxin transport or signaling reduced the growth-promoting and root developmental effects of inoculation [36], [113], [114]. It is likely that activating IAA signaling is required for microbe-induced plant growth and root development under saline stress, but definitive evidence is still lacking.

Ethylene is undoubtedly an important phytohormone involved in regulating plant growth under stress, but its role in plant salt tolerance is controversial [115]. Producing ACC deaminase, which is encoded by *acdS,* is an important characteristic of plant salt tolerance-inducing microbes [9]. ACC deaminase degrades ACC, the ethylene precursor, thereby reducing ethylene production and suppressing ethylene signaling, resulting in the activation of auxin signaling [9]. The significance of ACC deaminase-producing microbes in elevating salt tolerance has been recognized in *canola*, *wheat*, *tomato*, *barley* and *red pepper* [116], [117], [118], [119]. This function is evident based on the deficiency in decreasing plant ethylene under saline stress of the bacterial *acdS* mutant and on the enhanced salt-stress tolerance and reduced ethylene content in the acdS transgenic line of *Camelina sativa* [120] and rice [121]. In another example of microbes influencing ethylene signaling, *Enterobacter* sp. SA187 produces 2-keto-4-methylthiobutyric acid (KMBA), which can be converted into ethylene in planta, as well as ACC. de Zélicourt et al. [54] demonstrated that KMBA is responsible for modulating the plant ethylene signaling pathway and the SA187-induced salt tolerance of Arabidopsis. They deployed the pEBF2::GUS reporter in Arabidopsis and found that SA187 inoculation activated the expression of EBF2 in root tips as well as ACC treatment. By using several *Arabidopsis* mutants, de Zélicourt et al. [54] proposed that both SA187- and KMBA-induced salt tolerance is dependent on ethylene signaling rather than ethylene production or JA and ABA signaling. Conclusively, it seems that ACC deaminase and KMBA have somewhat similar effects on downstream ethylene signaling in different ways. Interestingly, de Zélicourt et al. [54] demonstrated that 100 nM ACC during salt stress could largely mimic the beneficial activity of SA187 on *Arabidopsis*, which should be opposite to the effect of ACC deaminase. Due to the controversial role of ethylene in plant abiotic stress tolerance, further investigation of the signaling perception in the ethylene signaling pathway upon ACC treatment, ACC deaminase and KMBA is needed to clarify the contradictory phenomenon.

Recently, two research groups simultaneously increased the interaction between microbe-induced plant salt tolerance and plant immunity. Loo et al. [122] proposed that microbe-associated molecular patterns (MAMPs) responsible for plant pattern-triggered immunity (PTI) could induce Arabidopsis salt tolerance when sensed by pattern-recognizing receptors (PRRs), a mechanism termed pattern-triggered salt tolerance (PTST). Rolli et al. [123] demonstrated that LYK4, which is responsible for the plant perception of chitin that triggers PTI, mediates *Enterobacter* sp.-triggered salt tolerance in *Arabidopsis thaliana*. These studies help to make sense of the crosstalk between microbe-induced biotic stress resistance and microbe-induced abiotic stress tolerance.

## 4 Summary and perspective

Due to the requirements of sustainable development and the multifunctional property of rhizosphere microbes, developing a microbial inoculant-based strategy is a promising direction for crop cultivation in saline soil. In recent years, scientists have drawn much attention to the isolation of strains and their effects on ion/osmotic balance, cell damage prevention and growth promotion of plants under saline stress. However, several issues remain that restrict the microbial strategy from taking further steps as a solution for enhancing crop tolerance in saline land.

1) The microbe-produced signaling molecules responsible for inducing plant salt tolerance are still lacking and remain to be identified. The identification of these molecules may support the development of chemical agents for promoting production in saline soil.
2) The detailed mechanisms by which microbes enhance plant salt tolerance, which is important for agricultural production, need to be deeply investigated. For example, many publications have demonstrated the regulation of plant NHXs or HKTs by microbes, but the underlying mechanisms are not clear. The mechanisms pertaining to the regulation of photosynthesis, antioxidants, osmolytes and growth by microbes are also not clear.
3) Some microbial species show efficiency in helping with plant tolerance to salt stress but are difficult to industrialize due to their relatively weak adaptability to either the production environment, storage environment, or saline soil environment. Therefore, matched technologies for microbial agent production and application need to be developed.
4) Enhancing crop production in saline soil is the final scope of research on microbe-induced plant salt tolerance, but there is still a lack of reports on the effect of microbial application on crop production in large-scale field experiments in saline soil, especially for some typical large saline soil land areas. The effects of microbes on crop production in different kinds of saline land may vary due to other factors, such as climate and soil type.

## Funding

This work was funded by the National Key Research and Development Program (2021YFF1000404), the Central Public-interest Scientific Institution Basal Research Fund (No. Y2022QC15), the Agricultural Science and Technology Innovation Program (ASTIP No. CAAS-ZDRW202201) and the TaiShan Industrial Experts Program (2018TSCYCX-37).

## CRediT authorship contribution statement

**Yunpeng Liu:** Funding acquisition, Writing – original draft. **Weibing Xun:** Writing- review and editing. **Lin Chen:** Visualization, Writing – original draft. **Haichao Feng:** Validation. **Zhihui Xu:** Writing- review& editing. **Nan Zhang:** Writing- review & editing. **Qiang Zhang:** Validation. **Ruifu Zhang:** Conceptualization.

## Declaration of Competing Interest

The authors declare that they have no known competing financial interests or personal relationships that could have appeared to influence the work reported in this paper.
