# Supplementary material for: Rhizosphere microbes enhance plant salt tolerance: Toward crop production in saline soil
Source: Comput Struct Biotechnol J. 2022 Nov 25;20:6543–51. doi: 10.1016/j.csbj.2022.11.046 (PMC9712829; doi:10.1016/j.csbj.2022.11.046)
Supplement: Supplementary data 1 [file mmc1.docx]

Table S1. Microbial strains capable to enhance plant salt-tolerance.

| **Microbes** | **Plant** | **EC or NaCl** | **Reference** |
| --- | --- | --- | --- |
| *AMF* |  |  |  |
| *Glomus fasciculatum* (Thaxter sensu Gerd.) Gerd. and Trappe | *Acacia Nilotica* | 9.5 dS/m | [1] |
| *Funneliformis mosseae, Rhizophagus irregularis* | *Arundo Donax* | 200 mM NaCl | [2] |
| *Rhizophagus irregularis* BGC BJ109 | *Black Locust* | 200 mM NaCl | [3] |
| *Rhizophagus irregularis* | *Cajanus Cajan* | 100mM NaCl | [4] |
| *Funneliformis mosseae, Rhizophagus irregularis* | *Cajanus Cajan* | 100 mM NaCl | [5] |
| *Funneliformis mosseae, Diversispora versiformis* | *Chrysanthemum Morifolium* | 50 mM NaCl | [6] |
| *Rhizophagus irregularis, Funneliformis mosseae* | *Cleopatra Mandarin* | 50 mM NaCl | [7] |
| *Claroideoglomus etunicatum, Rhizophagus intraradices, Funneliformis mosseae* | *Cucumber* | 200 mM NaCl | [8] |
| *Funneliformis mosseae, Septoglomus deserticola, Acaulospora lacunosa* | *Eclipta Prostrata* | 100 mM NaCl | [9] |
| *Glomus* sp.2*, Gigaspora albida, Gigaspora decipiens* | *Eucalyptus* | 20 dS/m | [10] |
| *Rhizophagus intraradices* (Schenck & Smith) | *Euonymus Maackii Rupr.* | 150 mM NaCl | [11] |
| *Glomus intraradices* (Schenck and Smith) | *Fenugreek* | 200 mM NaCl | [12] |
| *Rhizophagus intraradices, Funneliformis mosseae* | *Giant Reed* | 75 mM NaCl | [13] |
| *Funneliformis mosseae, Claroideoglomus lamellosum* | *Lettuce* | 60 mM NaCl | [14] |
| *Glomus intraradices* DAOM 197198 | *Lettuce* | 50 mM NaCl | [15] |
| *Glomus intraradices* (Schenck and Smith) strain EEZ 58 | *Lettuce* | 80 mM NaCl | [16] |
| *Glomus iranicum* var. *tenuihypharum* sp. nova | *Lettuce* | 4.19 dS/m | [17] |
| *Glomus intraradices* BAFC 3108 | *Lotus Glaber* | 200 mM NaCl | [18] |
| *Rhizophagus intraradices, Septoglomus constrictum, Claroideoglomus etunicatum* | *Maize* | 100 mM NaCl | [19,20] |
| *Glomus mosseae* | *Maize* | 0.2% NaCl | [21] |
| *Glomus mosseae* (Nicol. & Gerd.) Gerdemann & Trappe | *Maize* | 100 mM NaCl | [22] |
| *Glomus mosseae* NO.1 | *Maize* | 0.2% NaCl | [23] |
| *Glomus mosseae, Glomus intraradices*, *Glomus claroideum* | *Olive Trees* | 0.6% NaCl | [24] |
| *Funnelliformis mosseae* BEG12*, Acaulospora laevis* BEG13*, Gigaspora margarita* BEG34 | *Rice* | 120 mM NaCl | [25] |
| *Rhizoglomus fasciculatum, Gigaspora* sp. | *Pea* | Unknown | [26] |
| *Funneliformis mosseae; Rhizophagus intraradices, Claroideoglomus etunicatum* | *Sesbania Sesban* | 150 mM NaCl | [27] |
| *Glomus* sp. OGS12 | *Sorghum* | 200 mM NaCl | [28] |
| *Glomus etunicatum* Becker and *Gerdemann*, strain Sh21 | *Soybean* | 100 mM NaCl | [29] |
| *Glomus deserticola* | *Sweet Basil* | 10 dS/m | [30] |
| *Glomus mosseae* (Gerd. and Trappe) | *Tomato* | 7.4 dS/m | [31] |
| *Glomus intraradices, Glomus mosseae* | *Wheat* | 13 dS/m | [32] |
| *Rhizophagus irregularis* BEG140*, R. irregularis, Funneliformis mosseae* BEG95*, F. geosporum, Claroideoglomus claroideum* | *Wheat Plants* | 200 mM NaCl | [33] |
| *Funneliformis mosseae* BGC YN05 | *Zelkova Serrata* | 150 mM NaCl | [34] |
| *Bacillus* |  |  |  |
| *Bacillus subtilis* BERA 71 | *Acacia gerrardii* | 250 mM NaCl | [35] |
| *Bacillus* sp. L*, Sphingobacteriaceae* sp. K, *hizobium* sp. Y | *Arabidopsis* | 100 mM NaCl | [36] |
| *Bacillus oryzicola* YC7007 | *Arabidopsis* | 100 mM NaCl | [37] |
| *Bacillus amyloliquefaciens* FZB42 | *Arabidopsis* | 100 mM NaCl | [38] |
| *Bacillus subtilis* GB03 | *Arabidopsis* | 100 mM NaCl | [39] |
| *Pantoea stewartii* JZ2, JZ29*, Arthrobacter* JZ12*; Bacillus* sp. JZ34*, Microbacterium barkeri* JZ37 | *Arabidopsis* | 100mM NaCl | [40] |
| *Bacillus* sp. 1.SG.7, 5.SG.3, *Pseudomonas* sp. 2.SG.20, 2.C.19 | *Arabidopsis* | 150 mM NaCl | [41] |
| *Bacillus thuriengenesis* NEB 17 | *Arabidopsis and soybean* | 250 mM and 100 mM NaCl | [42,43] |
| *Bacillus mojavensis* S1; *Bacillus pumilus* S2; *Pseudomonas fluorescen* S3 | *Barley* | 200 mM NaCl | [44] |
| *Bacillus fortis* strain SSB21 | *Capsicum Annum* L. | 0.2% NaCl | [45] |
| *Bacillus subtilis* strain NUU4 | *Chickpea* | 2% NaCl | [46] |
| *Bacillus licheniformis* SA03 | *Chrysanthemum* | 50 mM NaHCO_3_ and 50 mM Na_2_CO_3_ | [47,48] |
| *Bacillus* sp. ST4, ST20, *B. sonorensis* ST5, *B. cereus* ST6, *B. subtilis* ST15, *B. safensis* ST17, *B. paramycoides* ST18, *B. cereus* ST22, *B. tequilensis* ST25 | *Cotton* | 200 mM NaCl or 10.51 ms/cm | [49] |
| *Bacillus amyloliquefaciens* | *Cotton and Okra* | 150 mM NaCl | [50] |
| *Bacillus* sp., *B. paramycoides* CO8 | *French Bean* | 100 mM NaCl | [51] |
| *Bacillus* sp., *Halobacillus* sp. | *Groundnut* | 1% NaCl | [52] |
| *Bacillus flexus* KLBMP 4941 | *Limonium sinense* | 200 mM NaCl | [53] |
| *Bacillus* sp. | *Maize* | 100 mM NaCl | [54] |
| *Bacillus megaterium* | *Maize* | 2.59 dS/m | [55] |
| *Bacillus amyloliquefaciens* SQR9 | *Maize,Arabidopsis* | 100 mM NaCl | [56,57] |
| *Bacillus amyloliquefaciens* SN13 | *Oryza Sativa* | 200 mM NaCl | [58–60] |
| *Bacillus pumilus* EU921259 | *Paddy* | 1.5% NaCl | [61] |
| *Paenibacillus yonginensis* DCY84T | *Panax ginseng Meyer* | 300 mM NaCl | [62] |
| *Bacillus pumilus* G5 | *Pharbitis Nil* | 50 mM NaCl | [63] |
| *Bacillus subtilis* 10-4, 26D | *Phaseolus Vulgaris* | 2% NaCl | [64] |
| *Bacillus subtilis* GB03 | *Puccinellia Tenuiflor* | 200 mM NaCl | [65] |
| *Bacillus licheniformis* QA1, *Enterobacter asburiae* QF11 | *Quinoa* | 400 mM NaCl | [66] |
| *Bacillus* sp. MN54 | *Quinoa* | 400 mM NaCl | [67] |
| *Bacillus* sp. | *Rice* | 100 mM NaCl | [68] |
| *Bacillus aryabhattai* MS3 | *Rice* | 200 mM NaCl | [69] |
| *Bacillus megaterium* JVBH4 | *Rice* | 1% NaCl | [70] |
| *Bacillus endophyticus, B. tequilensis* | *Salicornia Europaea* | 500 mM NaCl | [71] |
| *Bacillus megaterium* AK4, *B. aryabhattai* AK5 | *Soybean* | 200 mM NaCl | [72] |
| *Bacillus aryabhattai* ALT29 | *Soybean* | 240 mM NaCl | [73] |
| *Bacillus safensis* MF-01, *Bacillus altitudinis* MF-15, *Bacillus velezensis* MF-08 | *Maize* | Saline–Sodic Soil | [74] |
| *Bacillus mesonae* H20-5, *B. aryabhattai* H19-1 | *Tomato* | 36.5 dS/m | [75] |
| *Bacillus spizizenii* FMH45 | *Tomato* | 1% NaCl | [76] |
| *Bacillus velezensis FMH2* | *Tomato* | 171 mM NaCl | [77] |
| *Bacillus* sp. V62, V39, V1 | *Tomato* | 12 dS/m | [78] |
| *Arthrobacter* sp. TF1, TF7, *Bacillus megaterium* TF2, TF3 | *Tomato* | 300 mM NaCl | [79] |
| *Bacillus toyonensis* COPE52 | *Tomato* | 100 mM NaCl | [80] |
| *Bacillus* sp. EGY05, EGY21, EGY25 | *Tomato* | 200 mM NaCl | [81,82] |
| *Bacillus licheniformis* HSW-16 | *Wheat* | 200 mM NaCl | [83] |
| *Bacillus aquimaris* | *Wheat* | 5.2 dS/m | [84] |
| *Bacillus subtilis* LDR2 | *Wheat* | 100 mM NaCl | [85] |
| *Bacillus subtilis* MA17 | *Wheat* | 125 mM NaCl | [86] |
| *Bacillus methylotrophicus* PM19 | *Wheat* | 16 dS/m | [87] |
| *Bacillus frigoritolerans, B. thuringiensis, B. velezensis* | *Wheat* | 400 mM NaCl | [88] |
| *Bacillus subtilis* HG-15 | *Wheat* | 0.35% NaCl | [89] |
| *Bacillus subtilis* GB03 | *White Clover* | 150 mM NaCl | [90] |
| *Enterobacter* |  |  |  |
| *Enterobacter* sp. SA187 | *Arabidopsis* | 7.81 dS/m or 100 mM NaCl | [91] |
| *Enterobacter* sp. EJ01 | *Arabidopsis and Tomato* | 200 mM NaCl | [92] |
| *Enterobacter aerogenes* S14 | *Maize* | 12 dS/m | [93] |
| *Enterobacter* sp. | *Maize* | 13.6 dS/m | [94] |
| *Enterobacter bugandensis* SS3, *Enterobacter* sp. SS2 | *Mung Bean* | 1% NaCl | [95] |
| *Enterobacter* sp. UPMR18 | *Okra* | 75 mM NaCl | [96] |
| *Enterobacter* sp. MN17 | *Quinoa* | 400 mM NaCl | [67] |
| *Enterobacter tabaci* SA3 | *Rice* | 150 mM NaCl | [97] |
| *Enterobacter* sp. P23 | *Rice* | 150 mM NaCl | [98] |
| *Enterobacter cloacae* ZNP-4 | *Wheat* | 200 mM NaCl | [99] |
| *Piriformospora indica* |  |  |  |
| *P. indica* | *Arabidopsis* | 150 mM NaCl | [100] |
| *P. indica* | *Arabidopsis* | 200 mM NaCl | [101] |
| *P. indica* | *Barley* | 300 mM NaCl | [102] |
| *P. indica* | *Rice* | 200 mM NaCl | [103] |
| *P. indica* | *Rice* | 300 mM NaCl | [104] |
| *P. indica* | *Tomato* | 150 mM NaCl | [105] |
| *Pseudomonas* |  |  |  |
| *Pseudomonas putida* MTCC 5279 | *Arabidopsis* | 200 mM NaCl | [106] |
| *Pseudomonas migulae* 8R6 | *Camelina Sativa* | 20 dS/m | [107] |
| *Pseudomonas putida* KT2440 | *Citrus Macrophylla* | 90 mM NaCl | [108]. |
| *Pseudomonas putida* Rs-198 | *Cotton* | 0.35% NaCl | [109], |
| *Pseudomonas thivervalensis* SC5 | *Cucumber* | 100 mM NaCl | [110] |
| *Pseudomonas syringae* S5, *P. fluorescens* s20 | *Maize* | 12 dS/m | [93] |
| *Pseudomonas fluorescens* | *Maize* | 13.6 dS/m | [94] |
| *Pseudomonas geniculate* MF-84 | *Maize* | 150 mM NaCl | [111] |
| *Pseudomonas corrugata* CHM3 | *Oats and Barley* | 9.4 dS/m | [112] |
| *Pseudomonas sp.* MBE05, *P. stutzeri* MBE04 | *Peanut* | 100 mM NaCl | [113] |
| *Pseudomonas* RS-198, *Azospirillum brasilense* RS-SP7 | *Rapeseed* | 10 dS/m | [114] |
| *Pseudomonas simiae* AU | *Soybean* | 100mM NaCl | [115,116] |
| *Pseudomonas* sp. OFT5 | *Tomato* | 75 mM NaCl | [117] |
| *Pseudomonas fluorescens* YsS6, *P. migulae* 8R6 | *Tomato* | 185 mM NaCl | [118] |
| *Pseudomonas putida* UW4 | *Tomato* | 90 mM NaCl | [119,120] |
| *Pseudomonas chlororaphis* 30-84 | *Wheat* | 200 mM NaCl | [121] |
| *Pseudomona extremorientalis* TSAU6, *P. extremorientalis* TSAU20, *P. aurantiaca* TSAU22 | *Wheat Seed* | 100 mM NaCl | [122] |
| *Pseudomonas entomophila* PE3 | *sunflower* | 15.9 dS/m | [123] |
| *Pseudomonas azotoformans* CHB 1107 | *tomato* | 6 dS/m | [124] |
| *Trichoderma virens* 29.8 , *Trichoderma atroviride* IMI 206040 | *Arabidopsis* | 100 mM NaCl | [125] |
| *Trichoderma spp.* |  |  |  |
| *Trichoderma asperelloides* T203 | *Arabidopsis and Cucumber* | 100 mM NaCl | [126] |
| *Trichoderma harzianum* T-soybean | *Cucumber* | 200 mM NaCl | [127] |
| *Trichoderma asperellum* Q1 | *Cucumber* | 0.44% NaCl | [128] |
| *Trichoderma longibrachiatum* T6 | *Wheat* | 150 mM NaCl | [129] |
| *Trichoderma yunnanense* Th4, *T. afroharzianum* Th6 | *Wheat* | 200 mM NaCl | [130] |
| *Trichoderma harzianum* T-22 | *Barley* | 200 mM | [131] |
| *Trichoderma harzianum* T-22 | *Brassica juncea* | 200 mM | [132] |
| Others |  |  |  |
| *Aspergillus aculeatus* | *Bermudagrass* | 400 mM NaCl | [133] |
| *Aspergillus terreus* AL4 | *Pennisetum Glaucum* | 100mM NaCl | [134] |
| *Aspergillus terreus* AH1 | *Rice and Maize* | 150 mM NaCl | [135] |
| *Burkholderia phytofirmans* PsJN | *Arabidopsis* | 150 mM NaCl | [136] |
| *Burkholderia cepacia* SE4 | *Cucumber* | 120 mM NaCl | [137] |
| *Burkholderia phytofirmans* PsJN | *Quinoa* | 400 mM NaCl | [138] |
| *Burkholderia* sp. MTCC 12259 | *Rice* | 185 mM NaCl | [139] |
| *Paraburkholderia phytofirmans* PsJN | *Arabidopsis* | 150 mM NaCl | [140] |
| *Hypoxylon* sp. Sj18 | *Arabidopsis* | 300 mM NaCl | [141] |
| *Arthrobacter endophyticus* SYSU 333322, *Nocardiopsis alba* SYSU 333140 | *Arabidopsis* | 150 mM NaCl | [142] |
| *Stenotrophomonas maltophilia* BJ01 | *Peanut* | 100 mM NaCl | [143] |
| *Herbaspirillum* sp. GW103 | *Chinese Cabbage* | 150 mM NaCl | [144] |
| *Halomonas variabilis* HT1*, Planococcus rifietoensis* RT4 | *Chickpea* | 200 mM NaCl | [145] |
| *Pantoea dispersa* PSB3 | *Chickpea* | 150 mM NaCl | [146] |
| *Novosphingobium* sp. HR1a | *Alemow* | 90 mM NaCl | [147] |
| *Azospirillum brasilense* | *Corn* | 100 mM NaCl | [148] |
| *Klebsiella oxytoca* Rs-5 | *Cotton* | 0.7% NaCl | [149] |
| *Sordariomycetes* sp1-B’2 *and Melanconiella elegans*-21W2 | *Cowpea* | 5.0 dS/m | [150] |
| *Paecilomyces formosus* LHL10 | *Cucumber* | 120 mM NaCl | [151] |
| *Acinetobacter calcoaceticus* SE370*, Promicromonospora* sp. SE188 | *Cucumber* | 120 mM NaCl | [137] |
| *Aneurinibacillus aneurinilyticus* ACC02*; Paenibacillus* sp. ACC06 | *French Bean* | 2.5 dS/m | [152] |
| *Vibrio spartinae* | *Halimione portulacoides* | 510 mM NaCl | [153] |
| *Yarrowia lipolytica* FH1 | *Maize* | 100 mM NaCl | [154] |
| *Azotobacter strains* C5 | *Maize* | 100 mM NaCl | [155] |
| *Azotobacter chroococcum* 76A | *Tomato* | 100 mM NaCl | [156] |
| *Brevibacterium linens* RS16 | *Rice* | 100 mM NaCl | [157,158] |
| *Cronobacter sakazaki* SS5*, Cronobacter sakazaki* SS4 | *Mung Bean* | 1% NaCl | [95] |
| *Methylobacterium oryzae* CBMB20 | *Oryza Sativa* | 100 mM NaCl | [159] |
| *Gordonia* sp. JPA2 | *Pearl Millet* | 8 dS/m | [160] |
| *Azospirillum brasilense, Pantoea dispersa (together)* | *Pepper* | 120 mM NaCl | [161] |
| *Alcaligenes* sp. AF7 | *Rice* | 170 mM NaCl | [162] |
| *Myroides* sp. JIL321 | *Rice* | 150 mM NaCl | [163] |
| *Gordonia terrae* KMP456-M40 | *Rice and barley* | 5.6 dS/m for rice, 125 mM NaCl for barley | [164] |
| *Leclercia adecarboxylata* MO1 | *Solanum lycopersicum* | 120 mM NaCl | [165] |
| *Metarhizium anisopliae* LHL07 | *Soybean* | 140 mM NaCl | [151] |
| *Porostereum spadiceum* AGH786 | *Soybean* | 140 mM NaCl | [166] |
| *Micrococcus yunnanensis, Planococcus rifietoensis, Variovorax paradoxus* | *Sugar Beet* | 125 mM NaCl | [167] |
| *Sphingobacterium* BHU-AV3 | *Tomato* | 200 mM NaCl | [168] |
| *Achromobacter piechaudii* | *Tomato* | 172 mM NaCl | [169] |
| *Penicillium brevicompactum, Pseudomonas chrysogenum* | *Tomato and Lettuce* | 150 mM NaCl | [170] |
| *Staphylococcus sciuri* ET101 | *Tomato And Rice* | 400 mM NaCl | [171] |
| *Methylobacterium oryzae* CBMB20 | *Tomato Plant* | 100 mM NaCl | [172] |
| *Dietzia natronolimnaea* STR1 | *Wheat* | 150 mM NaCl | [85] |
| *Arthrobacter protophormiae* SA3 | *Wheat* | 100 mM NaCl | [85] |
| *Alternaria chlamydospora* | *Wheat* | 14 dS/m | [173] |
| *Klebsiella* sp. SBP-8 | *Wheat* | 200 mM NaCl | [174] |
| *Cronobacter sakazakii* OF115 | *Wheat* | 160 mM NaCl | [175] |
| *Streptomyces* sp. | *Maize* | 300 mM NaCl | [176] |
| *Rhizobium meliloti* strain Dormal | *Alfalfa* | 150 mM NaCl | [177] |
| *Rhizobium massiliae* KNUC7586 | *Pepper* | 200 mM NaCl | [178] |
| *Rhizobium* sp. SL42 | *Soybean* | 150 mM NaCl | [179] |
| *Streptomyces* sp. strains 2, *Microbacterium* sp. strain 4 | *Mesembryanthemum Crystallinum* | 200 mM NaCl | [180] |
| *Streptomyces venezuelae* ATCC 10712 | *Rice* | 150 mM NaCl | [181] |

References:

[1] Giri B, Kapoor R, Mukerji KG. Improved tolerance of *Acacia nilotica* to salt stress by arbuscular mycorrhiza, *Glomus fasciculatum* may be partly related to elevated K/Na ratios in root and shoot tissues. Microbial Ecology 2007;54:753–60. https://doi.org/10.1007/s00248-007-9239-9.

[2] Pollastri S, Savvides A, Pesando M, Lumini E, Volpe MG, Ozudogru EA, et al. Impact of two arbuscular mycorrhizal fungi on *Arundo donax* L. response to salt stress. Planta 2018;247:573–85. https://doi.org/10.1007/s00425-017-2808-3.

[3] Chen J, Zhang H, Zhang X, Tang M. Arbuscular mycorrhizal symbiosis alleviates salt stress in black locust through improved photosynthesis, water status, and K^+^/Na^+^ homeostasis. Frontiers in Plant Science 2017;8:1–14. https://doi.org/10.3389/fpls.2017.01739.

[4] Pandey R, Garg N. High effectiveness of *Rhizophagus irregularis* is linked to superior modulation of antioxidant defence mechanisms in *Cajanus cajan* (L.) Millsp. genotypes grown under salinity stress. Mycorrhiza 2017;27:669–82. https://doi.org/10.1007/s00572-017-0778-8.

[5] Garg N, Pandey R. Effectiveness of native and exotic arbuscular mycorrhizal fungi on nutrient uptake and ion homeostasis in salt-stressed *Cajanus cajan* L. (Millsp.) *Enotypes*. Mycorrhiza 2015;25:165–80. https://doi.org/10.1007/s00572-014-0600-9.

[6] Wang Y, Wang M, Li Y, Wu A, Huang J. Enhancements of arbuscular mycorrhizal fungi on growth and nitrogen acquisition of *Chrysanthemum morifolium* under salt stress. PLoS ONE 2017;35:36.

[7] Navarro JM, Pérez-Tornero O, Morte A. Alleviation of salt stress in citrus seedlings inoculated with arbuscular mycorrhizal fungi depends on the rootstock salt tolerance. Journal of Plant Physiology 2014;171:76–85. https://doi.org/10.1016/j.jplph.2013.06.006.

[8] Hashem A, Alqarawi AA, Radhakrishnan R, Al-Arjani ABF, Aldehaish HA, Egamberdieva D, et al. Arbuscular mycorrhizal fungi regulate the oxidative system, hormones and ionic equilibrium to trigger salt stress tolerance in Cucumis sativus L. Saudi Journal of Biological Sciences 2018;25:1102–14. https://doi.org/10.1016/j.sjbs.2018.03.009.

[9] Duc NH, Vo AT, Haddidi I, Daood H, Posta K. Arbuscular mycorrhizal fungi improve tolerance of the medicinal plant *Eclipta prostrata* (L.) and induce major changes in polyphenol profiles under salt stresses. Frontiers in Plant Science 2021;11:1–18. https://doi.org/10.3389/fpls.2020.612299.

[10] Klinsukon C, Lumyong S, Kuyper TW, Boonlue S. Colonization by arbuscular mycorrhizal fungi improves salinity tolerance of eucalyptus (*Eucalyptus camaldulensis*) seedlings. Scientific Reports 2021;11:1–10. https://doi.org/10.1038/s41598-021-84002-5.

[11] Li Z, Wu N, Meng S, Wu F, Liu T. Arbuscular mycorrhizal fungi (AMF) enhance the tolerance of *Euonymus maackii* Rupr. At a moderate level of salinity. PLoS ONE 2020;15:1–16. https://doi.org/10.1371/journal.pone.0231497.

[12] Evelin H, Giri B, Kapoor R. Ultrastructural evidence for AMF mediated salt stress mitigation in *Trigonella foenum*-graecum. Mycorrhiza 2013;23:71–86. https://doi.org/10.1007/s00572-012-0449-8.

[13] Romero-Munar A, Baraza E, Gulías J, Cabot C. Arbuscular mycorrhizal fungi confer salt tolerance in giant reed (*Arundo donax* L.) plants grown under low phosphorus by reducing leaf Na^+^ concentration and improving phosphorus use efficiency. Frontiers in Plant Science 2019;10:1–14. https://doi.org/10.3389/fpls.2019.00843.

[14] Santander C, Aroca R, Cartes P, Vidal G, Cornejo P. Aquaporins and cation transporters are differentially regulated by two arbuscular mycorrhizal fungi strains in lettuce cultivars growing under salinity conditions. Plant Physiology and Biochemistry 2021;158:396–409. https://doi.org/10.1016/j.plaphy.2020.11.025.

[15] Jahromi F, Aroca R, Porcel R, Ruiz-Lozano JM. Influence of salinity on the in vitro development of *Glomus intraradices* and on the in vivo physiological and molecular responses of mycorrhizal lettuce plants. Microbial Ecology 2008;55:45–53. https://doi.org/10.1007/s00248-007-9249-7.

[16] Aroca R, Ruiz-Lozano JM, Zamarreño ángel M, Paz JA, García-Mina JM, Pozo MJ, et al. Arbuscular mycorrhizal symbiosis influences strigolactone production under salinity and alleviates salt stress in lettuce plants. Journal of Plant Physiology 2013;170:47–55. https://doi.org/10.1016/j.jplph.2012.08.020.

[17] Vicente-Sánchez J, Nicolás E, Pedrero F, Alarcón JJ, Maestre-Valero JF, Fernández F. Arbuscular mycorrhizal symbiosis alleviates detrimental effects of saline reclaimed water in lettuce plants. Mycorrhiza 2014;24:339–48. https://doi.org/10.1007/s00572-013-0542-7.

[18] Sannazzaro AI, Echeverría M, Albertó EO, Ruiz OA, Menéndez AB. Modulation of polyamine balance in Lotus glaber by salinity and arbuscular mycorrhiza. Plant Physiology and Biochemistry 2007;45:39–46. https://doi.org/10.1016/j.plaphy.2006.12.008.

[19] Estrada B, Aroca R, Barea JM, Ruiz-Lozano JM. Native arbuscular mycorrhizal fungi isolated from a saline habitat improved maize antioxidant systems and plant tolerance to salinity. Plant Science 2013;201–202:42–51. https://doi.org/10.1016/j.plantsci.2012.11.009.

[20] Estrada B, Aroca R, Maathuis FJM, Barea JM, Ruiz-Lozano JM. Arbuscular mycorrhizal fungi native from a Mediterranean saline area enhance maize tolerance to salinity through improved ion homeostasis. Plant Cell and Environment 2013;36:1771–82. https://doi.org/10.1111/pce.12082.

[21] Sheng M, Tang M, Chen H, Yang B, Zhang F, Huang Y. Influence of arbuscular mycorrhizae on photosynthesis and water status of maize plants under salt stress. Mycorrhiza 2008;18:287–96. https://doi.org/10.1007/s00572-008-0180-7.

[22] Feng G, Zhang FS, Li XL, Tian CY, Tang C, Rengel Z. Improved tolerance of maize plants to salt stress by arbuscular mycorrhiza is related to higher accumulation of soluble sugars in roots. Mycorrhiza 2002;12:185–90. https://doi.org/10.1007/s00572-002-0170-0.

[23] Sheng M, Tang M, Chen H, Yang B, Zhang F, Huang Y. Influence of arbuscular mycorrhizae on the root system of maize plants under salt stress. Canadian Journal of Microbiology 2009;55:879–86. https://doi.org/10.1139/W09-031.

[24] Porras-Soriano A, Soriano-Martín ML, Porras-Piedra A, Azcón R. Arbuscular mycorrhizal fungi increased growth, nutrient uptake and tolerance to salinity in olive trees under nursery conditions. Journal of Plant Physiology 2009;166:1350–9. https://doi.org/10.1016/j.jplph.2009.02.010.

[25] Parvin S, Van Geel M, Yeasmin T, Verbruggen E, Honnay O. Effects of single and multiple species inocula of arbuscular mycorrhizal fungi on the salinity tolerance of a Bangladeshi rice (*Oryza sativa* L.) cultivar. Mycorrhiza 2020;30:431–44. https://doi.org/10.1007/s00572-020-00957-9.

[26] Parihar M, Rakshit A, Rana K, Tiwari G, Jatav SS. Arbuscular mycorrhizal fungi mediated salt tolerance by regulating antioxidant enzyme system, photosynthetic pathways and ionic equilibrium in pea (*Pisum sativum* L.). Biologia Futura 2020;71:289–300. https://doi.org/10.1007/s42977-020-00037-1.

[27] Abd-Allah EF, Hashem A, Alqarawi AA, Bahkali AH, Alwhibi MS. Enhancing growth performance and systemic acquired resistance of medicinal plant *Sesbania sesban* (L.) Merr using arbuscular mycorrhizal fungi under salt stress. Saudi Journal of Biological Sciences 2015;22:274–83. https://doi.org/10.1016/j.sjbs.2015.03.004.

[28] Yamato M, Ikeda S, Iwase K. Community of arbuscular mycorrhizal fungi in a coastal vegetation on Okinawa island and effect of the isolated fungi on growth of sorghum under salt-treated conditions. Mycorrhiza 2008;18:241–9. https://doi.org/10.1007/s00572-008-0177-2.

[29] Sharifi M, Ghorbanli M, Ebrahimzadeh H. Improved growth of salinity-stressed soybean after inoculation with salt pre-treated mycorrhizal fungi. Journal of Plant Physiology 2007;164:1144–51. https://doi.org/10.1016/j.jplph.2006.06.016.

[30] Elhindi KM, El-Din AS, Elgorban AM. The impact of arbuscular mycorrhizal fungi in mitigating salt-induced adverse effects in sweet basil (*Ocimum basilicum* L.). Saudi Journal of Biological Sciences 2017;24:170–9. https://doi.org/10.1016/j.sjbs.2016.02.010.

[31] Al-Karaki GN. Growth of mycorrhizal tomato and mineral acquisition under salt stress. Mycorrhiza 2000;10:51–4. https://doi.org/10.1007/s005720000055.

[32] Fileccia V, Ruisi P, Ingraffia R, Giambalvo D, Frenda AS, Martinelli F. Arbuscular mycorrhizal symbiosis mitigates the negative effects of salinity on durum wheat. PLoS ONE 2017;12:e0184158–e0184158. https://doi.org/10.1371/journal.pone.0184158.

[33] Eroğlu G, Cabral C, Ravnskov S, Bak Topbjerg H, Wollenweber B. Arbuscular mycorrhiza influences carbon-use efficiency and grain yield of wheat grown under pre- and post-anthesis salinity stress. Plant Biology 2020;22:863–71. https://doi.org/10.1111/plb.13123.

[34] Wang J, Zhai L, Ma J, Zhang J, Wang GG, Liu X, et al. Comparative physiological mechanisms of arbuscular mycorrhizal fungi in mitigating salt-induced adverse effects on leaves and roots of *Zelkova serrata*. Mycorrhiza 2020;30:341–55. https://doi.org/10.1007/s00572-020-00954-y.

[35] Hashem A, Abd Allah EF, Alqarawi AA, Al-Huqail AA, Shah MA. Induction of Osmoregulation and modulation of salt stress in *Acacia gerrardii* Benth. by arbuscular mycorrhizal fungi and *Bacillus subtilis* (BERA 71). Biomed Res Int 2016;2016:6294098. https://doi.org/10.1155/2016/6294098.

[36] Fan D, Subramanian S, Smith DL. Plant endophytes promote growth and alleviate salt stress in Arabidopsis thaliana. Scientific Reports 2020;10:1–18. https://doi.org/10.1038/s41598-020-69713-5.

[37] Baek D, Rokibuzzaman M, Khan A, Kim MC, Park HJ, Yun DJ, et al. Plant-growth promoting *Bacillus oryzicola* yc7007 modulates stress-response gene expression and provides protection from salt stress. Frontiers in Plant Science 2020;10:1–13. https://doi.org/10.3389/fpls.2019.01646.

[38] Liu S, Hao H, Lu X, Zhao X, Wang Y, Zhang Y, et al. Transcriptome profiling of genes involved in induced systemic salt tolerance conferred by *Bacillus amyloliquefaciens* FZB42 in Arabidopsis thaliana. Scientific Reports 2017;7:1–13. https://doi.org/10.1038/s41598-017-11308-8.

[39] Zhang H, Kim MS, Sun Y, Dowd SE, Shi H, Paré PW. Soil bacteria confer plant salt tolerance by tissue-specific regulation of the sodium transporter HKT1. Molecular Plant-Microbe Interactions 2008;21:737–44. https://doi.org/10.1094/MPMI-21-6-0737.

[40] Eida AA, Alzubaidy HS, de Zélicourt A, Synek L, Alsharif W, Lafi FF, et al. Phylogenetically diverse endophytic bacteria from desert plants induce transcriptional changes of tissue-specific ion transporters and salinity stress in *Arabidopsis thaliana*. Plant Science 2019;280:228–40. https://doi.org/10.1016/j.plantsci.2018.12.002.

[41] Venieraki A, Chorianopoulou SN, Katinakis P, Bouranis DL. Multi-trait wheat rhizobacteria from calcareous soil with biocontrol activity promote plant growth and mitigate salinity stress. Microorganisms 2021;9. https://doi.org/10.3390/microorganisms9081588.

[42] Subramanian S, Ricci E, Souleimanov A, Smith DL. A proteomic approach to lipo-chitooligosaccharide and thuricin 17 effects on soybean germinationunstressed and salt stress. PLoS ONE 2016;11:1–19. https://doi.org/10.1371/journal.pone.0160660.

[43] Subramanian S, Souleimanov A, Smith DL. Proteomic studies on the effects of lipo-chitooligosaccharide and thuricin 17 under unstressed and salt stressed conditions in *Arabidopsis thaliana*. Frontiers in Plant Science 2016;7:1–13. https://doi.org/10.3389/fpls.2016.01314.

[44] Mahmoud OMB, Slimene IB, Zribi OT, Abdelly C, Djébali N. Response to salt stress is modulated by growth-promoting rhizobacteria inoculation in two contrasting barley cultivars. Acta Physiologiae Plantarum 2017;39. https://doi.org/10.1007/s11738-017-2421-x.

[45] Yasin NA, Akram W, Khan WU, Ahmad SR, Ahmad A, Ali A. Halotolerant plant-growth promoting rhizobacteria modulate gene expression and osmolyte production to improve salinity tolerance and growth in *Capsicum annum* L. Environmental Science and Pollution Research 2018;25:23236–50. https://doi.org/10.1007/s11356-018-2381-8.

[46] Egamberdieva D, Wirth SJ, Shurigin VV, Hashem A, Abd Allah EF. Endophytic bacteria improve plant growth, symbiotic performance of chickpea (*Cicer arietinum* L.) and induce suppression of root rot caused by *Fusarium solani* under salt stress. Frontiers in Microbiology 2017;8:1–13. https://doi.org/10.3389/fmicb.2017.01887.

[47] Zhou C, Zhu L, Xie Y, Li F, Xiao X, Ma Z, et al. *Bacillus licheniformis* SA03 confers increased saline–alkaline tolerance in chrysanthemum plants by induction of abscisic acid accumulation. Frontiers in Plant Science 2017;8:1–17. https://doi.org/10.3389/fpls.2017.01143.

[48] Zhou C, Li F, Xie Y, Zhu L, Xiao X, Ma Z, et al. Involvement of abscisic acid in microbe-induced saline-alkaline resistance in plants. Plant Signaling & Behavior 2017;12:e1367465–e1367465. https://doi.org/10.1080/15592324.2017.1367465.

[49] Saleem S, Iqbal A, Ahmed F, Ahmad M. Phytobeneficial and salt stress mitigating efficacy of IAA producing salt tolerant strains in *Gossypium hirsutum*. Saudi Journal of Biological Sciences 2021;28:5317–24. https://doi.org/10.1016/j.sjbs.2021.05.056.

[50] Irizarry I, White JF. Application of bacteria from non-cultivated plants to promote growth, alter root architecture and alleviate salt stress of cotton. Journal of Applied Microbiology 2017;122:1110–20. https://doi.org/10.1111/jam.13414.

[51] Pandey S, Gupta S. Diversity analysis of ACC deaminase producing bacteria associated with rhizosphere of coconut tree (*Cocos nucifera* L.) grown in Lakshadweep islands of India and their ability to promote plant growth under saline conditions. Journal of Biotechnology 2020;324:183–97. https://doi.org/10.1016/j.jbiotec.2020.10.024.

[52] Banik A, Pandya P, Patel B, Rathod C, Dangar M. Characterization of halotolerant, pigmented, plant growth promoting bacteria of groundnut rhizosphere and its in-vitro evaluation of plant-microbe protocooperation to withstand salinity and metal stress. Science of the Total Environment 2018;630:231–42. https://doi.org/10.1016/j.scitotenv.2018.02.227.

[53] Xiong YW, Li XW, Wang TT, Gong Y, Zhang CM, Xing K, et al. Root exudates-driven rhizosphere recruitment of the plant growth-promoting rhizobacterium *Bacillus flexus* KLBMP 4941 and its growth-promoting effect on the coastal halophyte *Limonium sinense* under salt stress. Ecotoxicology and Environmental Safety 2020;194:110374–110374. https://doi.org/10.1016/j.ecoenv.2020.110374.

[54] Ullah S, Bano A. Isolation of plant-growth-promoting rhizobacteria from rhizospheric soil of halophytes and their impact on maize (*Zea mays* L.) under induced soil salinity. Canadian Journal of Microbiology 2015;61:307–13. https://doi.org/10.1139/cjm-2014-0668.

[55] Marulanda A, Azcón R, Chaumont F, Ruiz-Lozano JM, Aroca R. Regulation of plasma membrane aquaporins by inoculation with a Bacillus megaterium strain in maize (*Zea mays* L.) plants under unstressed and salt-stressed conditions. Planta 2010;232:533–43. https://doi.org/10.1007/s00425-010-1196-8.

[56] Chen L, Liu Y, Wu G, Veronican Njeri K, Shen Q, Zhang N, et al. Induced maize salt tolerance by rhizosphere inoculation of *Bacillus amyloliquefaciens* SQR9. Physiologia Plantarum 2016;158:34–44. https://doi.org/10.1111/ppl.12441.

[57] Chen L, Liu Y, Wu G, Zhang N, Shen Q, Zhang R. Beneficial rhizobacterium *Bacillus amyloliquefaciens* sqr9 induces plant salt tolerance through spermidine production. Molecular Plant-Microbe Interactions 2017;30:423–32. https://doi.org/10.1094/MPMI-02-17-0027-R.

[58] Nautiyal CS, Srivastava S, Chauhan PS, Seem K, Mishra A, Sopory SK. Plant growth-promoting bacteria *Bacillus amyloliquefaciens* NBRISN13 modulates gene expression profile of leaf and rhizosphere community in rice during salt stress. Plant Physiology and Biochemistry 2013;66:1–9. https://doi.org/10.1016/j.plaphy.2013.01.020.

[59] Chauhan PS, Lata C, Tiwari S, Chauhan AS, Mishra SK, Agrawal L, et al. Transcriptional alterations reveal *Bacillus amyloliquefaciens*-rice cooperation under salt stress. Scientific Reports 2019;9:1–13. https://doi.org/10.1038/s41598-019-48309-8.

[60] Tiwari S, Prasad V, Chauhan PS, Lata C. Bacillus amyloliquefaciens confers tolerance to various abiotic stresses and modulates plant response to phytohormones through osmoprotection and gene expression regulation in rice. Frontiers in Plant Science 2017;8:1–13. https://doi.org/10.3389/fpls.2017.01510.

[61] Jha Y, Subramanian RB. PGPR regulate caspase-like activity, programmed cell death, and antioxidant enzyme activity in paddy under salinity. Physiology and Molecular Biology of Plants 2014;20:201–7. https://doi.org/10.1007/s12298-014-0224-8.

[62] Sukweenadhi J, Balusamy SR, Kim YJ, Lee CH, Kim YJ, Koh SC, et al. A growth-promoting bacteria, *Paenibacillus yonginensis* DCY84T enhanced salt stress tolerance by activating defense-related systems in panax ginseng. Frontiers in Plant Science 2018;9:1–17. https://doi.org/10.3389/fpls.2018.00813.

[63] Zheng L, Ma X, Lang D, Zhang X, Zhou L, Wang L, et al. Encapsulation of *Bacillus pumilus* G5 from polyvinyl alcohol‑sodium alginate (PVA-SA) and its implications in improving plant growth and soil fertility under drought and salt soil conditions. International Journal of Biological Macromolecules 2022;209:231–43. https://doi.org/10.1016/j.ijbiomac.2022.04.017.

[64] Lastochkina O, Aliniaeifard S, Garshina D, Garipova S, Pusenkova L, Allagulova C, et al. Seed priming with endophytic *Bacillus subtilis* strain-specifically improves growth of *Phaseolus vulgaris* plants under normal and salinity conditions and exerts anti-stress effect through induced lignin deposition in roots and decreased oxidative and osmotic damages. Journal of Plant Physiology 2021;263:153462–153462. https://doi.org/10.1016/j.jplph.2021.153462.

[65] Niu SQ, Li HR, Paré PW, Aziz M, Wang SM, Shi H, et al. Induced growth promotion and higher salt tolerance in the halophyte grass *Puccinellia tenuiflora* by beneficial rhizobacteria. Plant and Soil 2016;407:217–30. https://doi.org/10.1007/s11104-015-2767-z.

[66] Mahdi I, Fahsi N, Hafidi M, Allaoui A, Biskri L. Plant growth enhancement using rhizospheric halotolerant phosphate solubilizing bacterium *Bacillus licheniformis* qa1 and *Enterobacter asburiae* qf11 isolated from chenopodium quinoa willd. Microorganisms 2020;8:1–21. https://doi.org/10.3390/microorganisms8060948.

[67] Yang A, Akhtar SS, Iqbal S, Amjad M, Naveed M, Zahir ZA, et al. Enhancing salt tolerance in quinoa by halotolerant bacterial inoculation. Functional Plant Biology 2016;43:632–42. https://doi.org/10.1071/FP15265.

[68] Misra S, Dixit VK, Khan MH, Kumar Mishra S, Dviwedi G, Yadav S, et al. Exploitation of agro-climatic environment for selection of 1-aminocyclopropane-1-carboxylic acid (ACC) deaminase producing salt tolerant indigenous plant growth promoting rhizobacteria. Microbiological Research 2017;205:25–34. https://doi.org/10.1016/j.micres.2017.08.007.

[69] Sultana S, Paul SC, Parveen S, Alam S, Rahman N, Jannat B, et al. Isolation and identification of salt-tolerant plant-growth-promoting rhizobacteria and their application for rice cultivation under salt stress. Canadian Journal of Microbiology 2020;66:144–60. https://doi.org/10.1139/cjm-2019-0323.

[70] Prittesh P, Avnika P, Kinjal P, Jinal HN, Sakthivel K, Amaresan N. Amelioration effect of salt-tolerant plant growth-promoting bacteria on growth and physiological properties of rice (*Oryza sativa*) under salt-stressed conditions. Archives of Microbiology 2020;202:2419–28. https://doi.org/10.1007/s00203-020-01962-4.

[71] Zhao Q, Yang XY, Li Y, Liu F, Cao XY, Jia ZH, et al. N-3-oxo-hexanoyl-homoserine lactone, a bacterial quorum sensing signal, enhances salt tolerance in *Arabidopsis* and wheat. Botanical Studies 2020;61. https://doi.org/10.1186/s40529-020-00283-5.

[72] Khan MA, Asaf S, Khan AL, Adhikari A, Jan R, Ali S, et al. Halotolerant rhizobacterial strains mitigate the adverse effects of NaCl stress in soybean seedlings. BioMed Research International 2019;2019. https://doi.org/10.1155/2019/9530963.

[73] Khan MA, Sahile AA, Jan R, Asaf S, Hamayun M, Imran M, et al. Halotolerant bacteria mitigate the effects of salinity stress on soybean growth by regulating secondary metabolites and molecular responses. BMC Plant Biology 2021;21:1–15. https://doi.org/10.1186/s12870-021-02937-3.

[74] Singh UB, Malviya D, Singh S, Singh P, Ghatak A, Imran M, et al. Salt‐tolerant compatible microbial inoculants modulate physio‐biochemical responses enhance plant growth, zn biofortification and yield of wheat grown in saline‐sodic soil. International Journal of Environmental Research and Public Health 2021;18. https://doi.org/10.3390/ijerph18189936.

[75] Yoo SJ, Weon HY, Song J, Sang MK. Induced tolerance to salinity stress by halotolerant bacteria *Bacillus aryabhattai* H19-1 and *B. Mesonae* H20-5 in tomato plants. Journal of Microbiology and Biotechnology 2019;29:1124–36. https://doi.org/10.4014/jmb.1904.04026.

[76] Masmoudi F, Tounsi S, Dunlap CA, Trigui M. Endophytic halotolerant *Bacillus velezensis* FMH2 alleviates salt stress on tomato plants by improving plant growth and altering physiological and antioxidant responses. Plant Physiology and Biochemistry 2021;165:217–27. https://doi.org/10.1016/j.plaphy.2021.05.025.

[77] Masmoudi F, Tounsi S, Dunlap CA, Trigui M. Halotolerant *Bacillus spizizenii* FMH45 promoting growth, physiological, and antioxidant parameters of tomato plants exposed to salt stress. Plant Cell Reports 2021;40:1199–213. https://doi.org/10.1007/s00299-021-02702-8.

[78] Tchakounté GVT, Berger B, Patz S, Becker M, Fankem H, Taffouo VD, et al. Selected rhizosphere bacteria help tomato plants cope with combined phosphorus and salt stresses. Microorganisms 2020;8:1–16. https://doi.org/10.3390/microorganisms8111844.

[79] Fan P, Chen D, He Y, Zhou Q, Tian Y, Gao L. Alleviating salt stress in tomato seedlings using *Arthrobacter* and *Bacillus megaterium* isolated from the rhizosphere of wild plants grown on saline–alkaline lands. International Journal of Phytoremediation 2016;18:1113–21. https://doi.org/10.1080/15226514.2016.1183583.

[80] Rojas-Solis D, Vences-Guzmán MA, Sohlenkamp C, Santoyo G. *Bacillus toyonensis* cope52 modifies lipid and fatty acid composition, exhibits antifungal activity, and stimulates growth of tomato plants under saline conditions. Current Microbiology 2020;77:2735–44. https://doi.org/10.1007/s00284-020-02069-1.

[81] Abdelshafy Mohamad OA, Ma JB, Liu YH, Zhang D, Hua S, Bhute S, et al. Beneficial endophytic bacterial populations associated with medicinal plant thymus vulgaris alleviate salt stress and confer resistance to *Fusarium oxysporum*. Frontiers in Plant Science 2020;11:1–17. https://doi.org/10.3389/fpls.2020.00047.

[82] Mohamad OAA, Liu YH, Li L, Ma JB, Huang Y, Gao L, et al. Synergistic plant-microbe interactions between endophytic actinobacteria and their role in plant growth promotion and biological control of cotton under salt stress. Microorganisms 2022;10. https://doi.org/10.3390/microorganisms10050867.

[83] Singh RP, Jha PN. A halotolerant bacterium *Bacillus licheniformis* HSW-16 augments induced systemic tolerance to salt stress in wheat plant (*Triticum aestivum*). Frontiers in Plant Science 2016;7:1–18. https://doi.org/10.3389/fpls.2016.01890.

[84] Upadhyay SK, Singh DP. Effect of salt-tolerant plant growth-promoting rhizobacteria on wheat plants and soil health in a saline environment. Plant Biology 2015;17:288–93. https://doi.org/10.1111/plb.12173.

[85] Barnawal D, Bharti N, Pandey SS, Pandey A, Chanotiya CS, Kalra A. Plant growth-promoting rhizobacteria enhance wheat salt and drought stress tolerance by altering endogenous phytohormone levels and TaCTR1/TaDREB2 expression. Physiologia Plantarum 2017;161:502–14. https://doi.org/10.1111/ppl.12614.

[86] Brahim AH, Ali MB, Daoud L, Jlidi M, Akremi I, Hmani H, et al. Biopriming of durum wheat seeds with endophytic diazotrophic bacteria enhances tolerance to *Fusarium* head blight and salinity. Microorganisms 2022;10. https://doi.org/10.3390/microorganisms10050970.

[87] Amna, Ud Din B, Sarfraz S, Xia Y, Kamran MA, Javed MT, et al. Mechanistic elucidation of germination potential and growth of wheat inoculated with exopolysaccharide and ACC- deaminase producing Bacillus strains under induced salinity stress. Ecotoxicology and Environmental Safety 2019;183:109466–109466. https://doi.org/10.1016/j.ecoenv.2019.109466.

[88] Wang R, Wang C, Feng Q, Liou RM, Lin YF. Biological inoculant of salt-tolerant bacteria for plant growth stimulation under different saline soil conditions. Journal of Microbiology and Biotechnology 2021;31:398–407. https://doi.org/10.4014/JMB.2009.09032.

[89] Ji C, Tian H, Wang X, Song X, Ju R, Li H, et al. *Bacillus subtilis* HG-15, a halotolerant rhizoplane bacterium, promotes growth and salinity tolerance in wheat (*Triticum aestivum*). BioMed Research International 2022;2022:1–16. https://doi.org/10.1155/2022/9506227.

[90] Han QQ, Lü XP, Bai JP, Qiao Y, Paré PW, Wang SM, et al. Beneficial soil bacterium *Bacillus subtilis* (GB03) augments salt tolerance of white clover. Frontiers in Plant Science 2014;5:1–8. https://doi.org/10.3389/fpls.2014.00525.

[91] de Zélicourt A, Synek L, Saad MM, Alzubaidy H, Jalal R, Xie Y, et al. Ethylene induced plant stress tolerance by *Enterobacter* sp. SA187 is mediated by 2‐keto‐4‐methylthiobutyric acid production. PLoS Genetics 2018;14:1–28. https://doi.org/10.1371/journal.pgen.1007273.

[92] Kim K, Jang Y-J, Lee S-M, Oh B-T, Chae J-C, Lee K-J. Alleviation of salt stress by *Enterobacter* sp. EJ01 in tomato and *Arabidopsis* is accompanied by up-regulation of conserved salinity responsive factors in plants. Molecules and Cells 2014;37:109–17. https://doi.org/10.14348/molcells.2014.2239.

[93] Nadeem SM, Zahir ZA, Naveed M, Arshad M. Preliminary investigations on inducing salt tolerance in maize through inoculation with rhizobacteria containing ACC deaminase activity. Canadian Journal of Microbiology 2007;53:1141–9. https://doi.org/10.1139/W07-081.

[94] Nadeem SM, Zahir ZA, Naveed M, Arshad M. Rhizobacteria containing ACC-deaminase confer salt tolerance in maize grown on salt-affected fields. Canadian Journal of Microbiology 2009;55:1302–9. https://doi.org/10.1139/W09-092.

[95] Desai S, Mistry J, Shah F, Chandwani S, Amaresan N, Supriya NR. Salt-tolerant bacteria enhance the growth of mung bean (*Vigna radiata* L.) and uptake of nutrients, and mobilize sodium ions under salt stress condition. International Journal of Phytoremediation 2022;0:1–8. https://doi.org/10.1080/15226514.2022.2057419.

[96] Habib SH, Kausar H, Saud HM. Plant growth-promoting rhizobacteria enhance salinity stress tolerance in Okra through ROS-scavenging enzymes. BioMed Research International 2016;2016:1–10. https://doi.org/10.1155/2016/6284547.

[97] Khan MA, Asaf S, Khan AL, Adhikari A, Jan R, Ali S, et al. Plant growth-promoting endophytic bacteria augment growth and salinity tolerance in rice plants. Plant Biology 2020;22:850–62. https://doi.org/10.1111/plb.13124.

[98] Sarkar A, Ghosh PK, Pramanik K, Mitra S, Soren T, Pandey S, et al. A halotolerant *Enterobacter* sp. displaying ACC deaminase activity promotes rice seedling growth under salt stress. Research in Microbiology 2018;169:20–32. https://doi.org/10.1016/j.resmic.2017.08.005.

[99] Singh RP, Pandey DM, Jha PN, Ma Y. ACC deaminase producing rhizobacterium *Enterobacter cloacae* ZNP-4 enhance abiotic stress tolerance in wheat plant. PLoS ONE 2022;17:1–23. https://doi.org/10.1371/journal.pone.0267127.

[100] Abdelaziz ME, Kim D, Ali S, Fedoroff NV, Al-Babili S. The endophytic fungus *Piriformospora indica* enhances *Arabidopsis* *thaliana* growth and modulates Na^+^/K^+^ homeostasis under salt stress conditions. Plant Science 2017;263:107–15. https://doi.org/10.1016/j.plantsci.2017.07.006.

[101] Vahabi K, Dorcheh SK, Monajembashi S, Westermann M, Reichelt M, Falkenberg D, et al. Stress promotes *Arabidopsis* - *Piriformospora indica* interaction. Plant Signaling and Behavior 2016;11. https://doi.org/10.1080/15592324.2015.1136763.

[102] Waller F, Achatz B, Baltruschat H, Fodor J, Becker K, Fischer M, et al. The endophytic fungus *Piriformospora indica* reprograms barley to salt-stress tolerance, disease resistance, and higher yield. Proceedings of the National Academy of Sciences of the United States of America 2005;102:13386–91. https://doi.org/10.1073/pnas.0504423102.

[103] Jogawat A, Vadassery J, Verma N, Oelmüller R, Dua M, Nevo E, et al. PiHOG1, a stress regulator MAP kinase from the root endophyte fungus *Piriformospora indica*, confers salinity stress tolerance in rice plants. Scientific Reports 2016;6:1–15. https://doi.org/10.1038/srep36765.

[104] Jogawat A, Saha S, Bakshi M, Dayaman V, Kumar M, Dua M, et al. *Piriformospora indica* rescues growth diminution of rice seedlings during high salt stress. Plant Signaling and Behavior 2013;8:e26891–e26891. https://doi.org/10.4161/psb.26891.

[105] Ghorbani A, Razavi SM, Ghasemi Omran VO, Pirdashti H. *Piriformospora indica* inoculation alleviates the adverse effect of NaCl stress on growth, gas exchange and chlorophyll fluorescence in tomato (Solanum lycopersicum L.). Plant Biology 2018;20:729–36. https://doi.org/10.1111/plb.12717.

[106] Srivastava S, Srivastava S. Prescience of endogenous regulation in *Arabidopsis* thaliana by *Pseudomonas putida* MTCC 5279 under phosphate starved salinity stress condition. Scientific Reports 2020;10:1–15. https://doi.org/10.1038/s41598-020-62725-1.

[107] Heydarian Z, Gruber M, Glick BR, Hegedus DD. Gene expression patterns in roots of camelina sativa with enhanced salinity tolerance arising from inoculation of soil with plant growth promoting bacteria producing 1-aminocyclopropane-1-carboxylate deaminase or expression the corresponding acds gene. Frontiers in Microbiology 2018;9:1–15. https://doi.org/10.3389/fmicb.2018.01297.

[108] Costa-Gutierrez SB, Lami MJ, Santo MCCD, Zenoff AM, Vincent PA, Molina-Henares MA, et al. Plant growth promotion by *Pseudomonas putida* KT2440 under saline stress: role of eptA. Applied Microbiology and Biotechnology 2020;104:4577–92. https://doi.org/10.1007/s00253-020-10516-z.

[109] Yao L, Wu Z, Zheng Y, Kaleem I, Li C. Growth promotion and protection against salt stress by *Pseudomonas putida* Rs-198 on cotton. European Journal of Soil Biology 2010;46:49–54. https://doi.org/10.1016/j.ejsobi.2009.11.002.

[110] Nascimento FX, Urón P, Glick BR, Giachini A, Rossi MJ. Genomic analysis of the 1-aminocyclopropane-1-carboxylate deaminase-producing *Pseudomonas thivervalensis* sc5 reveals its multifaceted roles in soil and in beneficial interactions with plants. Frontiers in Microbiology 2021;12. https://doi.org/10.3389/fmicb.2021.752288.

[111] Singh S, Singh UB, Trivedi M, Sahu PK, Paul S, Paul D, et al. Seed biopriming with salt-tolerant endophytic pseudomonas geniculata-modulated biochemical responses provide ecological fitness in maize (*Zea mays* L.) grown in saline sodic soil. International Journal of Environmental Research and Public Health 2020;17. https://doi.org/10.3390/ijerph17010253.

[112] Chang P, Gerhardt KE, Huang XD, Yu XM, Glick BR, Gerwing PD, et al. Plant growth-promoting bacteria facilitate the growth of barley and oats in salt-impacted soil: implications for phytoremediation of saline soils. International Journal of Phytoremediation 2014;16:1133–47. https://doi.org/10.1080/15226514.2013.821447.

[113] Sharma S, Kulkarni J, Jha B. Halotolerant rhizobacteria promote growth and enhance salinity tolerance in peanut. Frontiers in Microbiology 2016;7:1–11. https://doi.org/10.3389/fmicb.2016.01600.

[114] Farhangi-Abriz S, Tavasolee A, Ghassemi-Golezani K, Torabian S, Monirifar H, Rahmani HA. Growth-promoting bacteria and natural regulators mitigate salt toxicity and improve rapeseed plant performance. Protoplasma 2020;257:1035–47. https://doi.org/10.1007/s00709-020-01493-1.

[115] Vaishnav A, Kumari S, Jain S, Varma A, Tuteja N, Choudhary DK. PGPR-mediated expression of salt tolerance gene in soybean through volatiles under sodium nitroprusside. Journal of Basic Microbiology 2016;56:1274–88. https://doi.org/10.1002/jobm.201600188.

[116] Vaishnav A, Kumari S, Jain S, Varma A, Choudhary DK. Putative bacterial volatile-mediated growth in soybean (*Glycine max* L. *Merrill*) and expression of induced proteins under salt stress. Journal of Applied Microbiology 2015;119:539–51. https://doi.org/10.1111/jam.12866.

[117] Win KT, Fukuyo T, Keiki O, Ohwaki Y. The ACC deaminase expressing endophyte *Pseudomonas* spp. enhances NaCl stress tolerance by reducing stress-related ethylene production, resulting in improved growth, photosynthetic performance, and ionic balance in tomato plants. Plant Physiology and Biochemistry 2018;127:599–607. https://doi.org/10.1016/j.plaphy.2018.04.038.

[118] Ali S, Charles TC, Glick BR. Amelioration of high salinity stress damage by plant growth-promoting bacterial endophytes that contain ACC deaminase. Plant Physiology and Biochemistry 2014;80:160–7. https://doi.org/10.1016/j.plaphy.2014.04.003.

[119] Del Carmen Orozco-Mosqueda M, Duan J, DiBernardo M, Zetter E, Campos-García J, Glick BR, et al. The production of ACC deaminase and trehalose by the plant growth promoting bacterium *Pseudomonas* sp. UW4 synergistically protect tomato plants against salt stress. Frontiers in Microbiology 2019;10:1–10. https://doi.org/10.3389/fmicb.2019.01392.

[120] Yan J, Smith MD, Glick BR, Liang Y. Effects of ACC deaminase containing rhizobacteria on plant growth and expression of Toc GTPases in tomato (*Solanum lycopersicum*) under salt stress. Botany 2014;92:775–81. https://doi.org/10.1139/cjb-2014-0038.

[121] Yuan P, Pan H, Boak EN, Pierson LS, Pierson EA. Phenazine-producing rhizobacteria promote plant growth and reduce redox and osmotic stress in wheat seedlings under saline conditions. Frontiers in Plant Science 2020;11:1–12. https://doi.org/10.3389/fpls.2020.575314.

[122] Egamberdieva D. Alleviation of salt stress by plant growth regulators and IAA producing bacteria in wheat. Acta Physiologiae Plantarum 2009;31:861–4. https://doi.org/10.1007/s11738-009-0297-0.

[123] Fatima T, Arora NK. *Pseudomonas entomophila* PE3 and its exopolysaccharides as biostimulants for enhancing growth, yield and tolerance responses of sunflower under saline conditions. Microbiological Research 2021;244:126671–126671. https://doi.org/10.1016/j.micres.2020.126671.

[124] Liu CH, Siew W, Hung YT, Jiang YT, Huang CH. 1-Aminocyclopropane-1-carboxylate (ACC) deaminase gene in *Pseudomonas azotoformans* is associated with the amelioration of salinity stress in tomato. Journal of Agricultural and Food Chemistry 2021;69:913–21. https://doi.org/10.1021/acs.jafc.0c05628.

[125] Contreras-Cornejo HA, Macías-Rodríguez L, Alfaro-Cuevas R, López-Bucio J. *Trichoderma* spp. improve growth of arabidopsis seedlings under salt stress through enhanced root development, osmolite production, and Na^+^ elimination through root exudates. Molecular Plant-Microbe Interactions 2014;27:503–14. https://doi.org/10.1094/MPMI-09-13-0265-R.

[126] Brotman Y, Landau U, Cuadros-Inostroza Á, Takayuki T, Fernie AR, Chet I, et al. *Trichoderma*-plant root colonization: escaping early plant defense responses and activation of the antioxidant machinery for saline stress tolerance. PLoS Pathogens 2013;9:e1003221–e1003221. https://doi.org/10.1371/journal.ppat.1003221.

[127] Zhang F, Wang Y, Liu C, Chen F, Ge H, Tian F, et al. *Trichoderma harzianum* mitigates salt stress in cucumber via multiple responses. Ecotoxicology and Environmental Safety 2019;170:436–45. https://doi.org/10.1016/j.ecoenv.2018.11.084.

[128] Qi W, Zhao L. Study of the siderophore-producing *Trichoderma asperellum* Q1 on cucumber growth promotion under salt stress. Journal of Basic Microbiology 2013;53:355–64. https://doi.org/10.1002/jobm.201200031.

[129] Zhang S, Gan Y, Xu B. Application of plant-growth-promoting fungi *Trichoderma longibrachiatum* T6 enhances tolerance of wheat to salt stress through improvement of antioxidative defense system and gene expression. Frontiers in Plant Science 2016;7:1–11. https://doi.org/10.3389/fpls.2016.01405.

[130] Oljira AM, Hussain T, Waghmode TR, Zhao H, Sun H, Liu X, et al. *Trichoderma* enhances net photosynthesis, water use efficiency, and growth of wheat (*Triticum aestivum* L.) under salt stress. Microorganisms 2020;8:1–19. https://doi.org/10.3390/microorganisms8101565.

[131] Gupta S, Smith PMC, Boughton BA, Rupasinghe TWT, Natera SHA, Roessner U. Inoculation of barley with *Trichoderma harzianum* T-22 modifies lipids and metabolites to improve salt tolerance. Journal of Experimental Botany 2021;72:7229–46. https://doi.org/10.1093/jxb/erab335.

[132] Ahmad P, Hashem A, Abd-Allah EF, Alqarawi AA, John R, Egamberdieva D, et al. Role of *Trichoderma harzianum* in mitigating NaCl stress in Indian mustard (*Brassica juncea* L.) through antioxidative defense system. Frontiers in Plant Science 2015;6:868–868. https://doi.org/10.3389/fpls.2015.00868.

[133] Xie Y, Han S, Li X, Amombo E, Fu J. Amelioration of salt stress on bermudagrass by the fungus aspergillus aculeatus. Molecular Plant-Microbe Interactions 2017;30:245–54. https://doi.org/10.1094/MPMI-12-16-0263-R.

[134] Khushdil F, Jan FG, Jan G, Hamayun M, Iqbal A, Hussain A, et al. Salt stress alleviation in *Pennisetum glaucum* through secondary metabolites modulation by *Aspergillus terreus* L. Plant Physiology and Biochemistry 2019;144:127–34. https://doi.org/10.1016/j.plaphy.2019.09.038.

[135] Siddiqui ZS, Wei X, Umar M, Abideen Z, Zulfiqar F, Chen J, et al. Scrutinizing the application of saline endophyte to enhance salt tolerance in rice and maize plants. Frontiers in Plant Science 2022;12:1–15. https://doi.org/10.3389/fpls.2021.770084.

[136] Pinedo I, Ledger T, Greve M, Poupin MJ. *Burkholderia phytofirmans* PsJN induces long-term metabolic and transcriptional changes involved in *Arabidopsis thaliana* salt tolerance. Frontiers in Plant Science 2015;6:1–17. https://doi.org/10.3389/fpls.2015.00466.

[137] Kang SM, Khan AL, Waqas M, You YH, Kim JH, Kim JG, et al. Plant growth-promoting rhizobacteria reduce adverse effects of salinity and osmotic stress by regulating phytohormones and antioxidants in *Cucumis sativus*. Journal of Plant Interactions 2014;9:673–82. https://doi.org/10.1080/17429145.2014.894587.

[138] Yang A, Akhtar SS, Fu Q, Naveed M, Iqbal S, Roitsch T, et al. *Burkholderia phytofirmans* PsJN stimulate growth and yield of quinoa under salinity stress. Plants 2020;9:672–672. https://doi.org/10.3390/plants9060672.

[139] Sarkar A, Pramanik K, Mitra S, Soren T, Maiti TK. Enhancement of growth and salt tolerance of rice seedlings by ACC deaminase-producing *Burkholderia* sp. MTCC 12259. Journal of Plant Physiology 2018;231:434–42. https://doi.org/10.1016/j.jplph.2018.10.010.

[140] Ledger T, Rojas S, Timmermann T, Pinedo I, Poupin MJ, Garrido T, et al. Volatile-mediated effects predominate in *Paraburkholderia phytofirmans* growth promotion and salt stress tolerance of *Arabidopsis thaliana*. Frontiers in Microbiology 2016;7:1–18. https://doi.org/10.3389/fmicb.2016.01838.

[141] Huang G, Jin Q, Peng H, Zhu T, Ye H. Effect of a fungus, Hypoxylon spp., on endophytes in the roots of Asparagus. FEMS Microbiology Letters 2019;366:1–7. https://doi.org/10.1093/femsle/fnz207.

[142] Dong ZY, Narsing Rao MP, Wang HF, Fang BZ, Liu YH, Li L, et al. Transcriptomic analysis of two endophytes involved in enhancing salt stress ability of *Arabidopsis thaliana*. Science of the Total Environment 2019;686:107–17. https://doi.org/10.1016/j.scitotenv.2019.05.483.

[143] Alexander A, Singh VK, Mishra A. Halotolerant PGPR *Stenotrophomonas maltophilia* bj01 induces salt tolerance by modulating physiology and biochemical activities of *Arachis hypogaea*. Frontiers in Microbiology 2020;11:1–12. https://doi.org/10.3389/fmicb.2020.568289.

[144] Lee GW, Lee KJ, Chae JC. *Herbaspirillum* sp. strain GW103 alleviates salt stress in *Brassica rapa* L. ssp. pekinensis. Protoplasma 2016;253:655–61. https://doi.org/10.1007/s00709-015-0872-8.

[145] Qurashi AW, Sabri AN. Bacterial exopolysaccharide and biofilm formation stimulate chickpea growth and soil aggregation under salt stress. Brazilian Journal of Microbiology 2012;43:1183–91. https://doi.org/10.1590/S1517-83822012000300046.

[146] Panwar M, Tewari R, Gulati A, Nayyar H. Indigenous salt-tolerant rhizobacterium *Pantoea dispersa* (PSB3) reduces sodium uptake and mitigates the effects of salt stress on growth and yield of chickpea. Acta Physiologiae Plantarum 2016;38. https://doi.org/10.1007/s11738-016-2284-6.

[147] Vives-Peris V, Gómez-Cadenas A, Pérez-Clemente RM. Salt stress alleviation in citrus plants by plant growth-promoting rhizobacteria *Pseudomonas putida* and *Novosphingobium* sp. Plant Cell Reports 2018;37:1557–69. https://doi.org/10.1007/s00299-018-2328-z.

[148] Checchio MV, de Cássia Alves R, de Oliveira KR, Moro GV, Santos DMM dos, Gratão PL. Enhancement of salt tolerance in corn using *Azospirillum brasilense*: an approach on antioxidant systems. Journal of Plant Research 2021;134:1279–89. https://doi.org/10.1007/s10265-021-01332-1.

[149] Wu Z, Peng Y, Guo L, Li C. Root colonization of encapsulated *Klebsiella oxytoca* rs-5 on cotton plants and its promoting growth performance under salinity stress. European Journal of Soil Biology 2014;60:81–7. https://doi.org/10.1016/j.ejsobi.2013.11.008.

[150] Farias GC, Nunes KG, Soares MA, de Siqueira KA, Lima WC, Neves ALR, et al. Dark septate endophytic fungi mitigate the effects of salt stress on cowpea plants. Brazilian Journal of Microbiology 2020;51:243–53. https://doi.org/10.1007/s42770-019-00173-4.

[151] Khan AL, Hamayun M, Khan SA, Kang SM, Shinwari ZK, Kamran M, et al. Pure culture of *Metarhizium anisopliae* LHL07 reprograms soybean to higher growth and mitigates salt stress. World Journal of Microbiology and Biotechnology 2012;28:1483–94. https://doi.org/10.1007/s11274-011-0950-9.

[152] Gupta S, Pandey S. ACC deaminase producing bacteria with multifarious plant growth promoting traits alleviates salinity stress in French Bean (*Phaseolus vulgaris*) plants. Frontiers in Microbiology 2019;10:1–17. https://doi.org/10.3389/fmicb.2019.01506.

[153] Mateos-Naranjo E, Jurado JL, Redondo-Gómez S, Pérez-Romero JA, Glick BR, Rodríguez-Llorente ID, et al. Uncovering PGPB *Vibrio spartinae* inoculation-triggered physiological mechanisms involved in the tolerance of *Halimione portulacoides* to NaCl excess. Plant Physiology and Biochemistry 2020;154:151–9. https://doi.org/10.1016/j.plaphy.2020.05.034.

[154] Gul Jan F, Hamayun M, Hussain A, Jan G, Iqbal A, Khan A, et al. An endophytic isolate of the fungus *Yarrowia lipolytica* produces metabolites that ameliorate the negative impact of salt stress on the physiology of maize. BMC Microbiology 2019;19:1–10. https://doi.org/10.1186/s12866-018-1374-6.

[155] Rojas-Tapias D, Moreno-Galván A, Pardo-Díaz S, Obando M, Rivera D, Bonilla R. Effect of inoculation with plant growth-promoting bacteria (PGPB) on amelioration of saline stress in maize (*Zea mays*). Applied Soil Ecology 2012;61:264–72. https://doi.org/10.1016/j.apsoil.2012.01.006.

[156] Van Oosten MJ, Di Stasio E, Cirillo V, Silletti S, Ventorino V, Pepe O, et al. Root inoculation with *Azotobacter chroococcum* 76A enhances tomato plants adaptation to salt stress under low N conditions. BMC Plant Biology 2018;18:1–12. https://doi.org/10.1186/s12870-018-1411-5.

[157] Chatterjee P, Samaddar S, Niinemets Ü, Sa TM. *Brevibacterium linens* RS16 confers salt tolerance to *Oryza sativa* genotypes by regulating antioxidant defense and H^+^ ATPase activity. Microbiological Research 2018;215:89–101. https://doi.org/10.1016/j.micres.2018.06.007.

[158] Chatterjee P, Kanagendran A, Samaddar S, Pazouki L, Sa TM, Niinemets Ü. Inoculation of *Brevibacterium linens* RS16 in *Oryza sativa* genotypes enhanced salinity resistance: Impacts on photosynthetic traits and foliar volatile emissions. Science of the Total Environment 2018;645:721–32. https://doi.org/10.1016/j.scitotenv.2018.07.187.

[159] Chatterjee P, Kanagendran A, Samaddar S, Pazouki L, Sa TM, Niinemets Ü. *Methylobacterium oryzae* CBMB20 influences photosynthetic traits, volatile emission and ethylene metabolism in *Oryza sativa* genotypes grown in salt stress conditions. Planta 2019;249:1903–19. https://doi.org/10.1007/s00425-019-03139-w.

[160] Kayasth M, Kumar V, Gera R. Gordonia sp.: a salt tolerant bacterial inoculant for growth promotion of pearl millet under saline soil conditions. 3 Biotech 2014;4:553–7. https://doi.org/10.1007/s13205-013-0178-5.

[161] Del Amor FM, Cuadra-Crespo P. Plant growth-promoting bacteria as a tool to improve salinity tolerance in sweet pepper. Functional Plant Biology 2012;39:82–90. https://doi.org/10.1071/FP11173.

[162] Fatima T, Mishra I, Verma R, Arora NK. Mechanisms of halotolerant plant growth promoting *Alcaligenes* sp. involved in salt tolerance and enhancement of the growth of rice under salinity stress. 3 Biotech 2020;10:1–12. https://doi.org/10.1007/s13205-020-02348-5.

[163] Wang G, Li B, Peng D, Zhao H, Lu M, Zhang L, et al. Combined application of H_2_S and a plant growth promoting strain JIL321 regulates photosynthetic efficacy, soil enzyme activity and growth-promotion in rice under salt stress. Microbiological Research 2022;256:126943–126943. https://doi.org/10.1016/j.micres.2021.126943.

[164] Soldan R, Mapelli F, Crotti E, Schnell S, Daffonchio D, Marasco R, et al. Bacterial endophytes of mangrove propagules elicit early establishment of the natural host and promote growth of cereal crops under salt stress. Microbiological Research 2019;223–225:33–43. https://doi.org/10.1016/j.micres.2019.03.008.

[165] Kang SM, Shahzad R, Bilal S, Khan AL, Park YG, Lee KE, et al. Indole-3-acetic-acid and ACC deaminase producing *Leclercia adecarboxylata* MO1 improves *Solanum lycopersicum* L. growth and salinity stress tolerance by endogenous secondary metabolites regulation. BMC Microbiology 2019;19:1–14. https://doi.org/10.1186/s12866-019-1450-6.

[166] Hamayun M, Hussain A, Khan SA, Kim HY, Khan AL, Waqas M, et al. Gibberellins producing endophytic fungus *Porostereum spadiceum* AGH786 rescues growth of salt affected soybean. Frontiers in Microbiology 2017;8:1–13. https://doi.org/10.3389/fmicb.2017.00686.

[167] Zhou N, Zhao S, Tian CY. Effect of halotolerant rhizobacteria isolated from halophytes on the growth of sugar beet (*Beta vulgaris* L.) under salt stress. FEMS Microbiology Letters 2017;364:1–8. https://doi.org/10.1093/femsle/fnx091.

[168] Vaishnav A, Singh J, Singh P, Rajput RS, Singh HB, Sarma BK. *Sphingobacterium* sp. bhu-av3 induces salt tolerance in tomato by enhancing antioxidant activities and energy metabolism. Frontiers in Microbiology 2020;11:1–13. https://doi.org/10.3389/fmicb.2020.00443.

[169] Mayak S, Tirosh T, Glick BR. Plant growth-promoting bacteria confer resistance in tomato plants to salt stress. Plant Physiology and Biochemistry 2004;42:565–72. https://doi.org/10.1016/j.plaphy.2004.05.009.

[170] Molina-Montenegro MA, Acuña-Rodríguez IS, Torres-Díaz C, Gundel PE, Dreyer I. Antarctic root endophytes improve physiological performance and yield in crops under salt stress by enhanced energy production and Na^+^ sequestration. Scientific Reports 2020;10:5819–5819. https://doi.org/10.1038/s41598-020-62544-4.

[171] Taj Z, Challabathula D. Protection of photosynthesis by halotolerant *Staphylococcus sciuri* et101 in tomato (*Lycoperiscon esculentum*) and rice (*Oryza sativa*) plants during salinity stress: possible interplay between carboxylation and oxygenation in stress mitigation. Frontiers in Microbiology 2021;11:1–24. https://doi.org/10.3389/fmicb.2020.547750.

[172] Chanratana M, Joe MM, Roy Choudhury A, Anandham R, Krishnamoorthy R, Kim K, et al. Physiological response of tomato plant to chitosan-immobilized aggregated *Methylobacterium oryzae* CBMB20 inoculation under salinity stress. 3 Biotech 2019;9:1–13. https://doi.org/10.1007/s13205-019-1923-1.

[173] Bouzouina M, Kouadria R, Lotmani B. Fungal endophytes alleviate salt stress in wheat in terms of growth, ion homeostasis and osmoregulation. Journal of Applied Microbiology 2021;130:913–25. https://doi.org/10.1111/jam.14804.

[174] Singh RP, Jha P, Jha PN. The plant-growth-promoting bacterium *Klebsiella* sp. SBP-8 confers induced systemic tolerance in wheat (*Triticum aestivum*) under salt stress. Journal of Plant Physiology 2015;184:57–67. https://doi.org/10.1016/j.jplph.2015.07.002.

[175] Afridi MS, Amna, Sumaira, Mahmood T, Salam A, Mukhtar T, et al. Induction of tolerance to salinity in wheat genotypes by plant growth promoting endophytes: Involvement of ACC deaminase and antioxidant enzymes. Plant Physiology and Biochemistry 2019;139:569–77. https://doi.org/10.1016/j.plaphy.2019.03.041.

[176] Nozari RM, Ortolan F, Astarita LV, Santarém ER. *Streptomyces* spp. enhance vegetative growth of maize plants under saline stress. Brazilian Journal of Microbiology 2021;52:1371–83. https://doi.org/10.1007/s42770-021-00480-9.

[177] Wang Y, Yang P, Zhou Y, Hu T, Zhang P, Wu Y. A proteomic approach to understand the impact of nodulation on salinity stress response in alfalfa (*Medicago sativa* L.). Plant Biology 2022;24:323–32. https://doi.org/10.1111/plb.13369.

[178] Hahm MS, Son JS, Hwang YJ, Kwon DK, Ghim SY. Alleviation of salt stress in pepper (*Capsicum annum* L.) plants by plant growth-promoting rhizobacteria. Journal of Microbiology and Biotechnology 2017;27:1790–7. https://doi.org/10.4014/jmb.1609.09042.

[179] Ilangumaran G, Subramanian S, Smith DL. Soybean leaf proteomic profile influenced by rhizobacteria under optimal and salt stress conditions. Frontiers in Plant Science 2022;13:1–15. https://doi.org/10.3389/fpls.2022.809906.

[180] Kataoka R, Akashi M, Taniguchi T, Kinose Y, Yaprak AE, Turgay OC. Metabolomics analyses reveal metabolites affected by plant growth–promoting endophytic bacteria in roots of the halophyte *Mesembryanthemum crystallinum*. International Journal of Molecular Sciences 2021;22:11813–11813. https://doi.org/10.3390/ijms222111813.

[181] Yoolong S, Kruasuwan W, Thanh Phạm HT, Jaemsaeng R, Jantasuriyarat C, Thamchaipenet A. Modulation of salt tolerance in Thai jasmine rice (*Oryza sativa* L. cv. KDML105) by *Streptomyces venezuelae* ATCC 10712 expressing ACC deaminase. Scientific Reports 2019;9:1–10. https://doi.org/10.1038/s41598-018-37987-5.
